# Role of Auxin and Nitrate Signaling in the Development of Root System Architecture

**DOI:** 10.3389/fpls.2021.690363

**Published:** 2021-11-11

**Authors:** Qi-Qi Hu, Jian-Qin Shu, Wen-Min Li, Guang-Zhi Wang

**Affiliations:** School of Pharmacy, Chengdu University of Traditional Chinese Medicine, Chengdu, China

**Keywords:** auxin, nitrate, root architecture, polar auxin transport, nitrate transporter, primary nitrate signaling pathway, systemic nitrate signaling pathway

## Abstract

The plant root is an important storage organ that stores indole-3-acetic acid (IAA) from the apical meristem, as well as nitrogen, which is obtained from the external environment. IAA and nitrogen act as signaling molecules that promote root growth to obtain further resources. Fluctuations in the distribution of nitrogen in the soil environment induce plants to develop a set of strategies that effectively improve nitrogen use efficiency. Auxin integrates the information regarding the nitrate status inside and outside the plant body to reasonably distribute resources and sustainably construct the plant root system. In this review, we focus on the main factors involved in the process of nitrate- and auxin-mediated regulation of root structure to better understand how the root system integrates the internal and external information and how this information is utilized to modify the root system architecture.

## Introduction

Plant roots integrate signals relating to the internal and external environment of the plant and provide a vascular system that delivers the necessary nutrients, water, and hormones to various organs and tissues for growth, development, and reproduction (Jing and Strader, 2019). Typically, the root system comprises two types of roots: primary roots (PRs), derived from an embryonic radicle; and secondary roots, including lateral roots (LRs) and adventitious roots (ARs), that develop postembryonically from pericycle cells of the existing mature roots and aerial tissues such as shoots, stems, and leaves (Nibau et al., 2008; Bellini et al., 2014).

Postembryonic root growth mainly occurs in the distal tip in a region known as the root apical meristem, which comprises a group of stem cells that surround a distinct central region, termed the quiescent center (QC; Van Den Berg et al., 1997; Benfey and Scheres, 2000; Clowes, 2010). The QC consists of four mitotically inactive cells that maintain the identities of the surrounding stem cells by inhibiting the differentiation of initial stem cells. At the mid-heart stage, initial stem cells commence asymmetric division to form specific cells. A small group of initial stem cells display a repetitive division pattern (generating one initial cell and one daughter cell), gather together to form the root pro-meristem, and subsequently activate radial division patterns in other files of the derived daughter cells (Dolan et al., 1993; Scheres et al., 1994). The radially organized cells are located in concentric cylinders that are established during embryogenesis and preserved during postembryonic root growth, and further differentiate into specific root cells (Dolan et al., 1993). The process of LR formation begins *via* asymmetric transverse divisions and periclinal and anticlinal divisions by pericycle founder cells of the PRs; consequently, LRs and PRs exhibit an analogous morphology (Dolan et al., 1993; Dubrovsky et al., 2001; Casimiro et al., 2003). The cellular origins of multiple tissue-derived ARs remain obscure; however, they are thought to be mostly related to the founder cells generated in the cambium, phloem, and pericycle (Haissig, 1974).

Asymmetric cell divisions and subsequent LR initiation, primordium development, emergence, and elongation are under genetic control, with auxin representing the major regulator of the abovementioned processes (Takatsuka and Umeda, 2014). The plant-specific GRAS family of transcription factors (TFs) SCARECROW (SCR) and SHORT ROOT (SHR) act in parallel with the APETALA2 (AP2)-domain TFs PLETHORA1 (PLT1) and PLT2 to define stem cell niches by organizing QC cells and maintaining the activities of root stem cells (Aida et al., 2004). Furthermore, an SHR-interacting protein, SHRUBBY (SHBY), has been identified as a key link between the SHR/SCR and PLT pathways in the regulation of PR stem cell maintenance and radial patterning (Koizumi and Gallagher, 2013). Concomitant with auxin accumulation in root tips, genes encoding cyclin-dependent kinases (CDKs), such as CDKB2;1, CDKB2;2, and CYCD6;1 (a D-type cyclin) that act downstream of the SHR and SCR network facilitate the asymmetric division of cortex/endodermis initial cells (Sozzani et al., 2010). Additionally, the dynamic distribution of auxin that spans the root meristem underlies *PLT* gene expression gradients, from high levels in the QC to low levels in the stele, which translates into differing and concentration-dependent cellular responses (Galinha et al., 2007).

The large root surface area represented by LRs and ARs can be optimized for nutrient acquisition by improving the ability of the roots to detect nutrient fluctuation signals in the external environment (Lopez-Bucio, 2003; Bisseling and Scheres, 2014). Mineral nutrition availability is an important parameter affecting secondary root development. Nitrate, the main source of nitrogen for plants, activates local and systemic signaling pathways within the root that further regulate cell division and cell differentiation processes, thus exerting a profound impact on root system architecture (RSA; Lopez-Bucio, 2003). Although healthy secondary roots represent a means for unlimited resource acquisition, restricted nitrogen availability severely constrains this resource exploration behavior. Moreover, auxin signaling plays a key role in the adaptation to internal nitrate fluctuations and is important for the precise regulation of root structures (Vidal et al., 2010). Accordingly, in this review, we focus on how auxin and nitrate signaling influences RSA.

## Auxin Synthesis and Transport

Auxin is generally synthesized in young aerial tissues, such as cotyledons and developing leaves; however, studies have shown that the *de novo* synthesis of indole-3-acetic acid (IAA), the predominant form of auxin, also occurs in roots (Ljung et al., 2005). IAA can be synthesized *via* either a tryptophan (Trp)-dependent or Trp-independent pathway (Mano and Nemoto, 2012; Korasick et al., 2013; Olatunji et al., 2017). The Trp-dependent pathway is divided into four branches according to the main intermediate metabolites, that is, indole-3-pyruvic acid (IPA; Stepanova et al., 2008; Tao et al., 2008; Yamada et al., 2009; Zhao, 2012), indole-3-acetamide (IAM; Pollmann et al., 2009), indole-3-acetaldoxime (IAOx; Hull et al., 2000; Mikkelsen et al., 2004; Nafisi et al., 2007), and tryptamine (TAM; Zhao et al., 2001; Tao et al., 2008). Although root-specific IAA biosynthesis remains active in plants that lack shoots, auxin levels are significantly reduced, suggesting that IAA transport from source (shoot) to sink (root) tissues is necessary for IAA concentration gradient formation in root tips (Ljung et al., 2005). Nevertheless, that the LR primordium density is increased in shoot-excised seedlings suggests that root-derived IAA generated from IAM plays a compensatory role, at least to some extent, in maintaining auxin levels (Swarup et al., 2008). However, Chen et al. (2014) demonstrated that root-derived auxin plays an indispensable role in normal root development. *YUCCA* (*YUC*) genes encode flavin-containing monooxygenases, the rate-limiting enzyme in the conversion of IPA to IAA (Cao et al., 2019). Accordingly, the PR length of the *yucQ* mutant (with five simultaneously inactivated *YUC* genes) is considerably reduced compared with that of its wild-type (WT) counterpart. This *yucQ* root phenotype can be rescued by the addition of auxin to the growth media or by expressing one of the five inactivated *YUC* genes in the root, but not in shoots (Chen et al., 2014). *WEAK ETHYLENE INSENSITIVE8* (*WEI8*) and *TRYPTOPHAN AMINOTRANSFERASE RELATED2* (*TAR2*) genes encode tryptophan aminotransferases, enzymes that catalyze the conversion of Trp to IPA in the first step of the IPA pathway (Stepanova et al., 2008). The grafting of WT shoots onto *wei8 tar2* double mutant roots did not prevent root meristem degeneration; however, in the reciprocal grafting experiment, double mutant shoots did not alter the phenotype of WT roots (Brumos et al., 2018). This implies that root-derived auxin is sufficient for maintaining root meristem activity, which challenges the classic view that most auxin is produced in apical tissues and is then transported to root tips *via* the phloem. However, Bhalerao et al. (2002) demonstrated that the removal of the shoot apical meristem inhibits LR emergence, but has little effect on LR initiation. Interestingly, the authors showed that LR primordium initiation is triggered through an IAA concentration gradient established in the root before a detectable leaf-derived auxin pulse reached the root tips (Bhalerao et al., 2002). In addition, the application of exogenous IAA to the shoots of *wei8 tar2* double mutants cannot prevent the degeneration of LR, PR, and AR meristems, even though auxin is still transported to the root tips to catalyze LR and AR formation. However, exogenous IAA application to roots helps to preserve root meristem activity in *wei8 tar2* seedlings, thereby further confirming that root-derived auxin is essential for maintaining stem cell function (Brumos et al., 2018). In *Arabidopsis*, repeated programmed cell death in the LR cap drives the regular release of root-derived auxin into the elongation zone (oscillation zone), which contributes to the spatiotemporal regulation of lateral organs (Xuan et al., 2016). IAA accumulation in the elongation zone triggers AUX/IAA degradation and the activation of AUXIN RESPONSE FACTORs (ARFs), leading to a peak in the expression levels of the auxin reporter *DIRECT REPEAT5* (*DR5*), a synthetic promoter construct that acts as a readout for auxin activity. However, ARF activation induces AUX/IAA transcription, thus repressing ARF and *DR5* promoter activities. ARF activation at regular intervals (i.e., oscillation frequency) and auxin responses, as measured by *DR5* signal intensity (i.e., oscillation amplitude), determine pre-branch site formation (Xuan et al., 2020).

In plants, two distinct but connected (*via* the phloem) transport systems enable auxin transport from the shoot to the root cap. A rapid and uncontrolled bulk flow of nutrients in the phloem carries most of the IAA from apical tissues to the root, while another slower and transporter protein-controlled cell-to-cell mechanism, known as polar auxin transport (PAT), redistributes auxin in a more precise manner (Petrasek and Friml, 2009). Auxin transporters localized to the plasma membrane include auxin influx proteins [e.g., AUXIN RESISTANT1 (AUX1) and LIKE-AUX1 (LAX) family members] and efflux proteins [e.g., PIN-FORMED (PIN) and ATP-BINDING CASSETTE B4 (ABCB)/P-GLYCOPROTEIN (PGP) family members; Gälweiler et al., 1998; Geisler and Murphy, 2006; Peret et al., 2012; Cho and Cho, 2013]. Non-protonated auxin molecules generally enter cells by passive diffusion. Due to a chemiosmotic H^+^ gradient and an outside-positive membrane potential between the neutral cytoplasm and the acidic apoplast, some auxin anions (IAA^−^) can be co-transported across the plasma membrane with protons (2H^+^) through AUX/LAX proteins. In the more neutral cytoplasm, IAA (p*K*a=4.75) exists almost exclusively in a de-protonated form that can exit the cell only through polar PIN or nonpolar ABCB efflux carriers. PAT results mainly from asymmetric (polar) positioning of PIN proteins. Rootward auxin flow is maintained by the action of basally localized PIN1, PIN3, and PIN7 in stele cells. In addition, laterally localized PIN3 and PIN7 in columella cells transport root- and shoot-derived auxin to the epidermis, where apically localized PIN2 transports shootward-bound auxin to the elongation zone; however, some laterally localized PINs (PIN1, PIN3, and PIN7) in endodermal cells recycle some auxin from external cell files back into the vascular system, resulting in the formation of a local auxin gradient or auxin maxima in pericycle cells, which triggers asymmetric cell division before LR initiation (Petrasek and Friml, 2009; Grieneisen et al., 2012; Marhavy et al., 2014; Adamowski and Friml, 2015). These polar routes are further supported by AUX1, which regulates LR development by facilitating IAA loading into the leaf protophloem, IAA unloading from columella cells, and the import of IAA into the developing LR primordium (LRP; Marchant et al., 2002).

## Auxin Signal Perception and Transmission

As IAA passes through the plasma membrane, the AUXIN-BINDING PROTEIN 1 (ABP1) receptor and nuclear receptors belonging to the TRANSPORT INHIBITOR RESPONSE1/AUXIN SIGNALING F-BOX (TIR1/AFB1–3) protein family recognize and bind to this intracellular hormone leading to the activation of downstream signaling cascades (Brown and Jones, 1994; Dharmasiri et al., 2005b). The inactivation of ABP1 can reportedly impair root meristem activity and stem cell maintenance, implying that ABP1 mediates root growth by controlling the mitotic activity of meristematic and stem cells (Tromas et al., 2009). ABP1 inactivation leads to decreased expression of D-type cyclin and/or increased accumulation of *RETINOBLASTOMA-RELATED* (*RBR*) mRNA levels, thereby disrupting the G1/S-phase transition and contributing to the arrest of cell division (Tromas et al., 2009). In root cells, following the binding of auxin to ABP1, the *Arabidopsis* SPIKE1 (SPK1) transmembrane protein activates Rho-like GTPase from plants 6 (ROP6) and its effector ROP interactive CRIB motif-containing protein 1 (RIC1), which inhibits PIN2 endocytosis by stabilizing actin filaments (Robert et al., 2010; Chen et al., 2012; Lin et al., 2012). The cycling of PIN proteins between the plasma membrane and endosomes regulates their activity, thereby contributing to the spatial distribution of auxin in roots and the modulation of root growth and LR formation. However, the role of ABP1 in auxin perception is controversial. Neither *abp1* mutant – *abp1-c1* and *abp1-TD1* (a 5-bp deletion and a T-DNA insertion in the first exon of *ABP1*, respectively) – displays any obvious developmental defect. In addition, the expression levels of a set of auxin-inducible genes (*IAA3*, *5*, *7*, *13*, *17*, *19*) were similar between WT and *abp1* plants irrespective of the presence of auxin (Gao et al., 2015). Furthermore, whether ABP1 and SKP1 directly interact remains to be determined (Lin et al., 2012).

The SCF^TIR1^ E3 ubiquitin ligase complex is composed of three subunits, namely, a SKP1-like protein (ASK1 or ASK2), a cullin protein (CUL1), and an F-box protein (TIR1; Ciechanover, 1998). The auxin receptor T1R1 recruits AUX/IAA transcriptional repressors to the SCF^TIR1^ complex for ubiquitination and degradation, thus acting as a positive regulator of auxin signaling (Maraschin Fdos et al., 2009). Auxin binds to both TIR1 and AUX/IAA proteins to stabilize the SCF^TIR1^-AUX/IAA interaction (Dharmasiri et al., 2005a). Gray et al. (2001) demonstrated that SCF^TIR1^ interacts with IAA7/AXR2 and IAA17/AXR3 in an auxin-dependent manner. Moreover, the twisted horseshoe-shaped fold formed by the TIR1 leucine-rich repeat (LRR) domain contains a myo-inositol hexakisphosphate 6 (InsP6) co-factor that binds to auxin/auxin analogs and AUX/IAA peptide substrates (Gray et al., 2001; Tan et al., 2007). Auxin signal transduction is mediated by the ubiquitination of AUX/IAA proteins recruited by the TIR1/AFB1-3 receptors. The *TIR1* and *AFB1*, *AFB2*, and *AFB3* genes have similar sequences and perform overlapping functions in plant embryogenesis and development (Dharmasiri et al., 2005b). The levels of activity of these four proteins are different, with TIR1 and AFB2 being the dominant auxin receptors in seedling roots. The *tir1-1 afb2* double mutant is more resistant to auxin than the *tir1-1* single mutant; however, the auxin response of *tir1 afb1*, *tir1 afb3*, and *tir1 afb1 afb3* mutants is similar to that of *tir1* seedlings, indicating that TIR1 and AFB2 contribute more than AFB1 and AFB3 to the auxin response of roots (Parry et al., 2009). The nuclear-localized TIR1 auxin receptor is necessary for LR formation (Arase et al., 2012). Both *tir1-1* and *afb2-3* single mutants produced 50% fewer ARs compared with WT plants, while an additive effect was observed with the *tir1-1 afb2-3* double mutant. TIR1 and AFB2 recruit and form a co-complex with at least three AUX/IAA proteins (IAA6, IAA9, and IAA17). This results in the release of ARF6 and ARF8, which control the expression of *GH3.3*, *GH3.5*, and *GH3.6*, thereby modulating jasmonic acid (JA) homeostasis and stimulating AR initiation (Lakehal et al., 2019).

MicroRNAs are short, endogenously expressed, non-translated RNA-RNA duplexes derived from stem-loop regions of longer genome-encoded RNA precursors (pri-miRNAs) that have been cleaved twice by the RNase III enzyme DICER-Like 1 (DCL1). Although they were first identified as regulators of the timing of postembryonic development in *Caenorhabditis elegans* in 1993 (Lee et al., 1993), it is now known that miRNAs are key components of multiple regulatory networks in plants as well as other organisms. In plants, miRNAs bind to the RNA-induced silencing complex (RISC) and cleave target mRNAs through sequence complementarity, resulting in the repression of target genes (Jones-Rhoades et al., 2006; Trevisan et al., 2012). For example, *miR167* targets *ARF6* and *8*, and *miR160* targets *ARF10*, *16*, and *17*, thus downregulating their transcriptional levels (Gutierrez et al., 2009), while *miR393* regulates TIR1 and AFB3 receptor activity (Parry et al., 2009). Exogenous IAA application induces *miR393b* expression in plants. The overexpression of *miR393* reduces *TIR1* transcript levels, consequently enhancing auxin resistance, whereas *TIR1* overexpression results in auxin sensitivity, suggesting that *miR393* is a negative regulator of auxin perception (Chen et al., 2011). In soybean (*Glycine max* L.), auxin receptors encoded by *GmTIR1*/*AFB3*, orthologs of *Arabidopsis TIR1*/*AFB3*, positively regulate rhizobia infection and nodule development. The knockdown mutant of soybean *miR393* shows a significant increase in rhizobia infection and nodule number resulting from the accumulation of *GmTIR1/AFB3* transcript levels (Cai et al., 2017). In *Arabidopsis*, the expression of *miR393* and *AFB3* is induced by N metabolites and nitrate, respectively (Vidal et al., 2010). These observations suggest that the integration of signals relating to internal and external N availability by the unique N-responsive *miR393*/*AFB3* module plays a role in auxin responses.

## Modulation of the RSA by Nitrate–Auxin Signaling

Nitrogen, mainly in the form of nitrate absorbed through the roots, is necessary for plants to complete their life cycle. Nitrate is not only a source of N but also an important signaling molecule with roles in many biological processes (Crawford, 1995). The regulation of RSA by nitrate requires the coordinated activity of local and systemic signaling pathways that communicate the plant N status to different tissues and organs (Alvarez et al., 2012).

**Figure 1:**
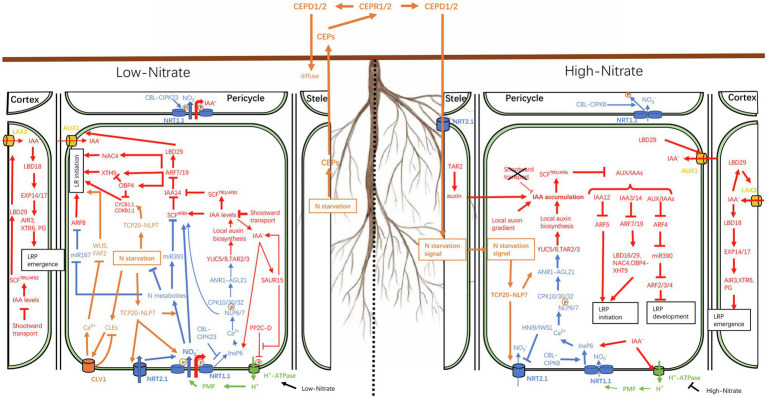
Schematic representation of the nitrate–auxin crosstalk under heterogeneous nitrate availability. When the external nitrate concentration is low (<0.05mM), phosphorylated NRT1.1, a high-affinity nitrate transporter, transports large amounts of nitrate into cells to alleviate the effects of internal nitrogen deficiency. NRT1.1, located on epidermal cells, also acts as an auxin transporter to facilitate shootward auxin transport, thereby reducing lateral auxin reflux and auxin accumulation in pericycle cells, resulting in the inhibition of lateral root (LR) initiation. When NRT1.1 is phosphorylated, its nitrate sensor function is inhibited. Consequently, the low level of local auxin biosynthesis, regulated by ANR1, cannot offset the loss of auxin. In addition, nitrate ions (NO_3_^−^) transported into cells can participate in the regulation of auxin-mediated LR formation through the *miR393-AFB3* module. When the external nitrate concentration is high (>10mM), NRT1.1 is dephosphorylated and functions mainly as a nitrate sensor to stimulate LR growth. The N starvation status signals of roots in low-NO_3_^−^ regions are transmitted to high-NO_3_^−^ regions through the CEP-CEPR1/2-CEPD1/2 module. Different from the demand for nitrate transport in low-NO_3_^−^ areas, in high-NO_3_^−^ areas, the TCP-NLP7 dimer is retained in the nucleus, where it acts to increase the sensitivity of the primary nitrate response (PNR). Lines ending in an inverted T (⊥) indicate negative regulation while line thickness indicates the strength of the pathways. Local nitrate, systemic nitrate, and auxin responses are shown in blue, yellow, and red, respectively. The protonmotive force (PMF) is shown in green.

The local nitrate response is also known as the primary nitrate response (PNR) as some genes involved in nitrate perception, assimilation, and transport respond to local nitrate stimulation within minutes, without *de novo* protein synthesis (Medici and Krouk, 2014). In addition to sensing local nitrate availability, plants must also integrate internal N demand signals of various regions to coordinate the overall resource allocation and regulate the growth and development of the root system, a process known as long-distance (or systemic) nitrate response (Forde, 2002). As a typical example, roots are known to preferentially colonize nitrate-rich soil areas in heterogeneous environments. Under conditions where one part of the root is exposed to low-nitrate conditions (<0.05mM) while another experiences high-nitrate availability (>10mM), roots in the nitrate-deficient patches may continue to signal nitrate deficiency to other regions, as if placing all roots in the same environment. This sustained signaling strengthens local nitrate sensitivity in the roots, which may explain why roots are well-developed in nitrate-rich patches (Figure 1). In this process, the distribution of auxin in root tissues responds to fluctuations in nitrate availability, which, in turn, affects the establishment of local auxin maxima or local auxin gradients, thus triggering a series of processes that modulate the RSA.

### Nitrate-IAA Crosstalk During Nitrate Uptake

#### Nitrate/IAA Absorption Driven by the Protonmotive Force

Nitrate entry into epidermal and cortical cells of the plant root system constitutes the first step in nitrogen acquisition in plants. This process is accomplished by membrane-localized nitrate transporters in a process that is driven by the hydrogen ion concentration gradient or protonmotive force (PMF) generated by plasma membrane H^+^-ATPases (PM H^+^-ATPases; Crawford, 1995). The proton chemical gradient and the outside-positive membrane potential generated by H^+^ efflux provide chemical and electrical energy for driving solute transport by other transporter systems and channels. Proton efflux also causes apoplast acidification, which promotes cell expansion (Haruta et al., 2015). The acid-growth hypothesis proposes that auxin-induced elongation may be related to plasma membrane hyperpolarization caused by auxin-mediated activation of PM H^+^-ATPases, which results in the acidification of the apoplast and the subsequent loosening of the cell wall, allowing cells to expand. In *Arabidopsis*, auxin activates H^+^-ATPase 2 (AHA2) by indirectly promoting the phosphorylation of the penultimate threonine residue (Thr947) within its carboxy-terminal regulatory domain *via* auxin-responsive SMALL AUXIN UP-RNAs (SAUR15/19; Spartz et al., 2014). Members of the protein phosphatase type 2C (PP2C-D) family prevent the activation of this enzyme through the dephosphorylation of Thr947; however, SAURs can bind to PP2C-D phosphatases and inhibit their enzymatic activity, suggesting that the activity of PM H^+^-ATPases is regulated by SAUR-PP2C-D modules (Spartz et al., 2014).

Recently, Yin et al. (2020) demonstrated that *Arabidopsis SAUR15* acts downstream of ARF6/8 and ARF7/19 to activate PM H^+^-ATPases, which drives cell expansion and facilitates LR and AR formation. In addition, SAUR15 can induce the local expression of auxin biosynthesis genes, such as *TAR4*, *YUC6*, *YUC7*, *ALDEHYDE OXIDASE1* (*AAO1*), *CYTOCHROME P450 FAMILY 71* (*CYP71A13*), and *NITRILASE 1* (*NIT1*), resulting in an increase in the free auxin concentration, thereby forming a positive feedback loop. Interestingly, the phosphorylation status of the H^+^-ATPases in the *tir1-1 afb2-3* and *axr1-3* mutants is similar to that of WT plants (Takahashi et al., 2012). Nonetheless, this does not mean that TIR1/AFBs are not involved in auxin-induced acid growth. A synthetic convex IAA (cvxIAA) developed by Uchida et al. (2018) only induces the expression of downstream auxin-responsive genes in seedlings expressing the engineered concave TIR1 (ccvTIR1) receptor. The cvxIAA elicits rapid SAUR19 expression, leading to increased levels of PM H^+^-ATPase phosphorylation in the hypocotyls of *TIR1pro::ccvTIR1 tir1 afb2* seedlings (Uchida et al., 2018). Combined, these observations indicate that the TIR1/AFB signaling pathway is involved in auxin-induced acid growth, but acts redundantly with other as yet unidentified pathways. ABP_57_, a 57-kDa auxin-binding receptor in rice, activates plant PM H^+^-ATPases *via* direct interaction. Exogenous IAA (5μM) treatment significantly enhances the affinity of ABP_57_ for PM H^+^-ATPases, which, in turn, leads to an increase in the H^+^-efflux efficiency of this enzyme (Kim et al., 2001; Lee et al., 2009). Although no ABP_57_ homolog has been found in *Arabidopsis* (Lee et al., 2009), it seems likely that some ABPs also play a role in the auxin-induced H^+^-ATPases phosphorylation and acid growth in this plant.

Inconsistent with the auxin-induced H^+^-ATPases phosphorylation, low-nitrate availability facilitates solute transport (potentially including NO_3_^−^ and IAA^−^) by increasing the level of transcription of the *AHA2* gene, thus promoting PR and LR development; however, the opposite is seen under high-nitrate conditions (Mlodzinska et al., 2015). Although there is no direct evidence to indicate that IAA or NO_3_^−^ can improve NO_3_^−^/IAA^−^ absorption efficiency by activating H^+^-ATPases, several studies have indicated that nitrate absorption efficiency might be regulated by auxin (Guo et al., 2002; Zheng et al., 2013).

#### Nitrate Transporters

Plants possess two sets of nitrate transport systems [a high-affinity transport system (HATS) and a low-affinity transport system (LATS)] to ensure optimal efficiency of nitrate uptake by roots. Large amounts of nitrate are stored in root vacuoles as the nitrogen pool or transferred to the shoots *via* the xylem. Some of the nitrate in the cytosol is reduced to nitrite by nitrate reductase (NR) and is then further reduced to ammonia by nitrite reductase (NiR) after entering the plastid (Crawford, 1995; Crawford and Glass, 1998). In the plastid, ammonia can be immobilized into amino acids (glutamine, glutamate, aspartate, and asparagine) by the activity of nitrogen assimilation enzymes such as glutamine synthetase (GS) and glutamate synthase (GOGAT; Lam et al., 1995).

##### The NRT1.1 Transceptor

The affinity of *Arabidopsis thaliana NRT1.1* (*AtNRT1.1*, also known as *CHL1*/*AtNPF6.3*), a dual-affinity nitrate transporter, for nitrate can be altered through the phosphorylation and dephosphorylation of Thr101 (located in the loop between domains 2 and 3) in response to fluctuations in nitrate availability (Liu and Tsay, 1999, 2003; Ho et al., 2009). Under low-nitrate or nitrate-deprivation conditions, CALCINEURIN B-LIKE proteins (CBLs), a subfamily of plant-specific Ca^2+^ sensors, activate CBL-INTERACTION PROTEIN KINASE 23 (CIPK23), which then phosphorylates Thr101 in NRT1.1, thereby decoupling the NRT1.1 dimer into monomers that have high structural flexibility and function as a high-affinity nitrate transporter to develop nitrogen use efficiency (NUE). In contrast, CIPK8 dephosphorylates Thr101, resulting in NRT1.1 dimerization, which converts it into a low-affinity transporter (Sun et al., 2014). In addition to its role in nitrate uptake, NRT1.1 also acts as a sensor in nitrate signaling (Gojon et al., 2011). Furthermore, as with nitrate uptake, the function of NRT1.1 as a sensor also shows a dual-affinity pattern. The phosphorylation and dephosphorylation of Thr101 lead to low- and high-level PNRs, respectively. The CBL-CIPK23 complex blocks the nitrate binding site of NRT1.1 *via* Thr101 phosphorylation, thus keeping nitrate signal transmission at a low level; however, Thr101 phosphorylation is necessary for maintaining high-affinity nitrate transport to alleviate internal N starvation in roots in low-nitrate patches (Ho et al., 2009). In contrast, the CBL/CIPK8 complex interferes with the nitrate transport function of NRT1.1 but exposes the nitrate binding site of NRT1.1, thus continuously triggering the nitrate response (Hu et al., 2009; Figure 1).

NRT1.1 also acts as an auxin transporter. Two hypotheses have been postulated to explain this NRT1.1-dependent auxin transport. On the one hand, NRT1.1 and AUX/LAX proteins both function as amino acid carriers and, therefore, the structural similarity between auxin and amino acids, such as Trp, supports the auxin transport properties of NRT1.1 (Williams and Miller, 2001). On the other hand, under low-nitrate availability, phosphorylated NRT1.1, acting as a high-affinity nitrate transporter, promotes nitrate transportation into cells, resulting in increased transcription levels of *AHA2* and the generation of a PMF to drive IAA transport. Conversely, dephosphorylated NRT1.1 inhibits IAA transport into cells (Mlodzinska et al., 2015).

NRT1.1 localization in epidermal and cortical cells facilitates shootward auxin transport under low-nitrate (<0.5mM) or N-free conditions, leading to low auxin concentrations in immature LR meristems. This, in turn, inhibits LR and AR development in a TIR1/AFB pathway-dependent manner (Table 1). Under high-nitrate conditions (>1mM), various processes associated with NRT1.1-mediated shootward auxin transport are inhibited (Krouk et al., 2010; Maghiaoui et al., 2020). NRT1.1-dependent shootward auxin transport inhibits lateral auxin reflux from epidermal to stele cells, thereby obstructing IAA accumulation in cortical and pericycle cells. Thus, IAA3/14-ARF7/19, IAA12-ARF5, and IAAs-ARF4 module-dependent LRP initiation and development are inhibited in pericycle cells. Moreover, the *LATERAL ORGAN BOUNDARIES DOMAIN 29* (*LBD29*) gene, acting downstream of the TIR1/AFB7/19 module, blocks AUX1 from transporting IAA into pericycle cells in response to low-nitrate availability (<0.5mM), while also affecting the function of the auxin influx carrier LAX3 located on cortical cells, thus hindering the formation of a local auxin gradient. However, the latter is necessary to initiate auxin responses to promote the expression of *LBD18* and downstream cell wall loosening/remodeling-associated genes (expansin, *EXP14/17*; subtilisin-like protease 7, *AIR3*; polygalacturonase, *PG*; xyloglucosyl transferase, *XTR6*) in cortical and epidermal cells that surround the LRP, resulting in cell separation, which aids LRP emergence (Krouk et al., 2010; Mounier et al., 2014; Maghiaoui et al., 2020). In addition, a reduction in LRP density also lowers the demand for local auxin supply from stele cells for LR development, leading to a decrease in the expression levels of the auxin biosynthesis gene *TAR2* in the endoplasmic reticulum (ER) of stele cells in regions of low-nitrate availability (Krouk et al., 2010; Lay-Pruitt and Takahashi, 2020).

**Table 1:** Summary of the role of ARF genes in the development of root system architecture (RSA).

Gene name	Plant species	Role in nitrate response?	Role in RSA development	References
***Negative regulators***				
*AtARF2* *AtARF3* *AtARF4*	*Arabidopsis thaliana*	NO	*TAS3a*, *miR390a*→ta-siRNAs⊥ARF2/3/4⊥LRP elongation	Marin et al., 2010; Yoon et al., 2010
			IAA→SCF^TIR1^⊥Aux/IAAs⊥ARF4⊥miR390a (negative feedback)ARF2/3→miR390a (positive feedback)	
			*AtARF10* *AtARF16*		*A. thaliana*	NO	IAA→SCF^TIR1/AFBs^⊥IAA17⊥ARF10/16⊥WOX5→PLT1 levels→maintain distal stem cell (DSC) activity	Wang et al., 2005; Ding and Friml, 2010; Ma et al., 2014
miR160⊥ARF10/16⊥WOX5→PLT1 levels→promote DSC differentiation→PR elongation								
*AtARF17*	*A. thaliana*	NO	IAA→SCF^TIR1/AFB2^⊥IAA6, IAA9, IAA17⊥ARF17⊥GH3.3, GH3.5, GH3.6⊥GH3.11 (JAR1)→jasmonoyl-L-isoleucine (JA-lle) formation⊥AR formation miR160⊥ARF17	Gutierrez et al., 2009, 2012; Lakehal et al., 2019
			***Positive regulators***				
*AtARF5*	*A. thaliana*	NO	IAA→SCF^TIR1/AFB2^⊥IAA12, IAA3⊥ARF5→initiate root meristem formation during embryogenesis	Hamann et al., 2002; Weijers et al., 2005; De Smet et al., 2010; Dastidar et al., 2019
		NO	IAA→SCF^TIR1/AFB2^⊥IAA12⊥ARF5→LRI	
*AtARF6* *AtARF8*	*A. thaliana*	NO	IAA→SCF^TIR1/AFB2^⊥IAA6, IAA9, IAA17 ⊥ARF6/8→GH3.3, GH3.5, GH3.6⊥GH3.11→JA-lle formation⊥AR formation miR167⊥ARF6/8	Gifford et al., 2008; Gutierrez et al., 2009, 2012; Lakehal et al., 2019
			YES		Low NO_3_^−^→NRT1.1 transporter→NO_3_^−^→N metabolites ⊥miR167⊥ARF8→LRI
		*AtARF7/19*	*A. thaliana*		YES	Low NO_3_^−^→NRT1.1 transporter→NO_3_^−^→N metabolites →miR393⊥SCF^AFB3^⊥IAA14⊥ARF7/19→NAC4, XHT9→LRI	Weijers et al., 2005; Okushima et al., 2007; Vidal et al., 2010; Windels and Vazquez, 2011; Feng et al., 2012; Goh et al., 2012a,b; Vidal et al., 2014; Chen et al., 2015; Porco et al., 2016; Xu et al., 2016; Lee et al., 2019; Xu and Cai, 2019
N metabolites →miR393⊥SCF^AFB3^⊥IAA14⊥ARF7/19→OBP4⊥XHT9→LRI							
N metabolites →miR393⊥SCF^AFB3^⊥IAA14⊥ARF7/19→OBP4⊥CYCB1;1, CDKB1;1→LRI							
Low NO_3_^−^→NRT1.1 transporter→NO_3_^−^→SCF^AFB3^							
NO	IAA→SCF^TIR1/AFBs^⊥IAA3⊥ARF7/19→LBD29→AUX1, LAX3→LRP initiation, LRP emergence, and AR initiationARF7/19⊥IAA3 (negative feedback)						
	NO			IAA→SCF^TIR1/AFBs^⊥IAA3⊥ARF7/19→LBD16, LBD18, LBD33→LR and AR initiation, LRP development, and LRP emergence			
NO	IAA→SCF^TIR1/AFBs^⊥IAA3⊥ARF7→MYB124 (FLP)→LRI						

LRP, lateral root primordium; ARs, adventitious roots; LRs, lateral roots; LRI, lateral root initiation; N, nitrogen; PRs, primary roots. ⊥: negative regulation; →: positive regulation.

Interestingly, under low-nitrate conditions (<0.5mM), *TAR2* expression is upregulated in pericycle and vascular tissues, leading to an increase in LR numbers (Ma et al., 2014). In addition, in response to low-nitrate availability, the expression of the *AGL21* gene (encoding a MADS-box TF) and the downstream auxin biosynthesis genes (*YUC5*, *YUC8*, and *TAR3*) is upregulated, leading to increased local auxin supply, which stimulates LRP germination and LR growth (Yu et al., 2014). This may be related to the sensor function of NRT1.1, which mobilizes PNRs to locally upregulate the expression of auxin synthesis genes. The PNR has more advantages under high-nitrate conditions than under low-nitrate conditions. Using a split-root experiment, Mounier et al. (2014) showed that NRT1.1 phosphorylation-dependent auxin shootward transport greatly reduces auxin accumulation in the LRP and young LR tips, which represses LRP initiation. On the high-nitrate side (>0.5mM), auxin shootward transport is inhibited, thereby allowing auxin accumulation, while the nitrate signaling pathway is activated locally, which stimulates LR elongation (Mounier et al., 2014; Figure 1). Therefore, the preferential proliferation of LRs in nitrate-rich patches under uneven NO_3_^−^ distribution is achieved through the “double” dual-functions (double nitrate/auxin transport, double nitrate response) of NRT1.1.

##### NRT2.1 Transporters

Barley (*Hordeum vulgare* L. cv. Midas) has a strong nitrate or ammonium absorption capacity within a few days after being transferred from a nitrate supply medium to a nitrogen-deficient environment, although there is no change in root growth. Moreover, during the first 3days of growth on nitrogen-deprived medium, even though the relative concentration of non-nitrate nitrogen decreased by 36%, the nitrate transport capacity increased, indicating that the nitrogen status of the plant parallels the effects of nitrogen deficiency on nitrate and ammonium absorption (Lee and Rudge, 1986). This compensatory upregulation of nitrate uptake in response to nitrogen deprivation was further verified in the split-root system of *Arabidopsis* and was found to be related to the high-affinity transporters NRT2.1 and NRT2.2 (Zhuo et al., 1999; Cerezo et al., 2001). The feedback regulation of nitrate absorption by nitrogen metabolites and the induction of HATS activity by nitrate deficiency were both suppressed in *atnrt2* plants (Cerezo et al., 2001). In addition, in the *lin1* mutant (substitution of a conserved amino acid in NRT2.1), the inhibitory effect of low external nitrate levels on LR initiation is alleviated, suggesting that NRT2.1 may be involved in the NRT1.1-dependent inhibition of LR initiation (Little et al., 2005).

Both external nitrate deficiency and N starvation activate the transport function of NRT2.1. Conversely, high-nitrate supply inhibits the expression of *NRT2.1*, an effect that is associated with HIGH NITROGEN INSENSITIVE 9 (HNI9)/INTERACT WITH SPT6 (IWS1), a component of the RNA polymerase II (RNAPII) complex, which mediates the upregulation of histone H3 lysine 27 trimethylation (H3K27me3) levels at the *NRT2.1* locus (Widiez et al., 2011). Interestingly, the N starvation-mediated feedback regulation of NRT2.1 transporter function is not limited to the N-deficient side in the split-root experiment, but also affects long-distance (shoot-to-root) nitrate signaling (Wang et al., 2018; Fredes et al., 2019). The C-terminally ENCODED PEPTIDES (CEPs) secreted by *Arabidopsis* roots in response to nitrogen starvation-related stimuli transmit nitrogen deficiency signals to the leaves through the xylem vessels (Tabata et al., 2014). The diffusion of CEPs to the surface of leaf phloem cells and their perception by two LRR-RKs – CEP RECEPTOR 1 and 2 (CEPR1/2) – lead to the secretion of two CEP DOWNSTREAM (CEPD) polypeptides (CEPD1 and CEPD2) that translocate to the roots in both high- and low-nitrate zones and activate nitrate responses (Tabata et al., 2014). Nonetheless, CEPDs scattered in all patches only activate the transporter function of NRT2.1 in roots where nitrate is available, suggesting that NRT2.1 may be involved in CEPD transport (Ohkubo et al., 2017).

### Nitrate-IAA Crosstalk in the Process of Nitrate Signaling Transmission

Upon perception, the nitrate signal is transmitted from the plasma membrane to the nucleus and then to downstream regulators. This signal transmission is mainly achieved through both Ca^2+^-dependent and Ca^2+^-independent pathways (Riveras et al., 2015).

#### The Ca^2+^-Dependent Pathway

In the Ca^2+^-dependent pathway, under nitrate stimulation, Ca^2+^ acts as a secondary messenger downstream of NRT1.1. In the presence of nitrate signals, the concentrations of inositol 1,4,5-trisphosphate (InsP3) and Ca^2+^ in the cytoplasm are increased in a NRT1.1-dependent manner; however, these effects are blocked by exposure to a pharmacological inhibitor of phospholipase C (PLC; U73122), but not by a nonfunctional PLC inhibitor analog (U73343; Riveras et al., 2015). In plants, PLCs, important membrane phospholipid-hydrolyzing enzymes in the phosphoinositide metabolic pathway, are subdivided into two categories according to the specificity of the hydrolyzed substrates, namely, phosphatidylinositol-PLC (PI-PLC) and phosphatidylcholine-PLC (PC-PLC)/non-specific PLC (NPC; Singh et al., 2015). PI-PLCs catalyze the hydrolysis of the precursor lipid phosphatidylinositol 4,5-bisphosphate [PtdIns(4,5)P_2_], yielding diacylglycerol (DAG) and InsP3. In mammals, InsP3 is released into the cytoplasm and binds to InsP3 receptors (InsP3-Rs), resulting in the release of Ca^2+^ from the ER (Singh et al., 2015; Berridge, 2016). This suggests that the PI-PLC-InsP3-Ca^2+^ pathway plays a key role in nitrate signaling. However, no gene corresponding to InsP3-Rs has been identified in plants, and the low quantity of PtdIns(4,5)P_2_ detected in higher plants seems to indicate that InsP3-induced Ca^2+^ release is not present in plants (Krinke et al., 2007; Van Leeuwen et al., 2007). Studies have shown that InsP3 is converted to InsP6 by inositol polyphosphate kinase 1 (IPK1) and IPK2 (Stevenson-Paulik et al., 2002). InsP6, a Ca^2+^-mobilizing agent, participates in signal transduction events by releasing Ca^2+^ from the vacuole and other endomembrane stores (Lemtiri et al., 2003). Given the involvement of InsP6 in auxin perception, it is possible that IAA mediates secondary signal (Ca^2+^) transmission in nitrate responses (Tan et al., 2007).

Functional targeted screening identified subgroup III Ca^2+^-sensor protein kinases (CPKs; CPK10, CPK30, and CPK32) as novel regulators of PNRs that enhance the expression of nitrate-responsive genes, even at very low-nitrate concentrations (Krouk, 2017; Liu et al., 2017). These three functionally redundant subgroup III enzymes act as the primary regulators of downward Ca^2+^ signal transmission. Concurrent with the increase in Ca^2+^ levels both inside and outside the nucleus in response to nitrate, subgroup III enzymes can rapidly facilitate nuclear transit and directly phosphorylate the Ser205 residue on NIN-Like Protein7 (NLP7), a member of the *Arabidopsis* NODULE INCEPTION (NIN)-like protein family. NLP7 is a master regulator of the PNR, positively regulating the development of RSA by post-translationally regulating downstream TFs and PNR-related genes (Castaings et al., 2009; Marchive et al., 2013; Krouk, 2017). Compared with WT seedlings, the activities of major nitrate–CPK target genes, as well as LRP density and LR elongation, were significantly inhibited in the “inducible *cpk10*,*30*,*32*” (*icpk*) triple mutant (Liu et al., 2017). Nevertheless, this does not imply that nitrate-stimulated root development is only regulated by the Ca^2+^-dependent pathway. The inductive effect of *AFB3* was significantly altered in *AtNRT1.1*–*5* and *AtNRT1.1*–*9* (*chl1*–*5* and *chl1*–*9*) mutants but remained unchanged in WT plants grown in the presence of the Ca^2+^ channel blocker lanthanum chloride (LaCl_3_) or U73122. This effect was different from that observed for other nitrate-responsive genes, suggestive of the existence of a Ca^2+^-independent pathway in nitrate signal transmission (Riveras et al., 2015).

#### The Ca^2+^-Independent Pathway

In the Ca^2+^-independent pathway, internal nitrate activates *AFB3*, which then coordinates PR and LR growth through a downstream regulatory network (Ulmasov et al., 1997; Vidal et al., 2010). AFB3, a member of the TIR1/AFB clade of auxin receptors (TAARs) in the AFB family of plant F-box proteins, degrades AUX/IAA transcriptional repressor proteins, thereby releasing ARF TFs that then initiate the primary auxin response (Si-Ammour et al., 2011). Additionally, a high-nitrate supply suppresses root development by reducing PR and LR meristem activity. Interestingly, these inhibitory effects are lost in *AFB3* mutants (*afb3-1*, *afb2-1*, and *afb1-1*), but are enhanced in NR-deficient mutants, suggesting that nitrate assimilation products regulate *AFB3* expression in response to nitrate *via* a feedback loop (Zhang et al., 1999; Vidal et al., 2010).

Recent studies have shown that *miR393* coordinates the expression of different sets of TAAR-encoding genes under various biotic or abiotic stresses (Lu et al., 2018). Vidal et al. (2010) showed that *miR393* negatively regulates *AFB3* mRNA levels in roots in response to nitrate and N metabolites generated by nitrate reduction and assimilation. This suggests that nitrate indirectly induces *miR393* to maintain intracellular AFB3 homeostasis through negative feedback regulation. This effect is in contrast to that seen with the ARF8-miR167 module, in which N metabolites inhibit the miR167-mediated degradation of *ARF8* mRNA, thus promoting LR initiation (Gifford et al., 2008).

### Nitrate-IAA Crosstalk in Local/Systemic Nitrate Pathways

#### NLP7

NLP7, one of the nine members of the *Arabidopsis* NIN-like family of RWP-RK TFs, acts as a master regulator of the early response to nitrate availability. The *Arabidopsis nlp7* mutant displays PNR response defects and a nitrogen starvation phenotype, including a lower shoot-to-root fresh weight ratio (S/R ratio), even in the presence of nitrate (Castaings et al., 2009). In response to nitrate signaling, AtNLP7 accumulates rapidly in the nucleus, while under nitrogen starvation conditions, NLP7-GFP localizes to the cytoplasm, but transfers to the nucleus within minutes of nitrate resupply (Marchive et al., 2013). The addition of the nuclear export inhibitor leptomycin B to *NLP7-GFP* seedlings grown in nitrogen-deprived medium resulted in partial NLP7 nuclear accumulation. This is consistent with the hypothesis that NLP7 accumulates rapidly in the nucleus due to the nitrate-mediated inhibition of the nuclear export-related receptor Exportin1 (XPO1/CRM1; Fornerod et al., 1997; Kudo et al., 1998; Marchive et al., 2013), an effect that allows a wide range of NLP7-dependent nuclear genes to be instantaneously mobilized in early nitrate responses.

The N-terminal region of NLP6 (the closest NLP7 homolog), which flanks the RWP-RK domain, receives the nitrate signal, which stimulates its conversion of NLP6 from an inactive to an active state, thus contributing to the positive regulation of the primary nitrate-responsive genes and TFs. The mechanism of activation may be related to the CPK-mediated phosphorylation of Ser205 located in the N-terminal region of NLP7. This candidate CPK phosphorylation site, identified through the alignment of nine *Arabidopsis* NLPs with four orthologous *Lotus japonicus* NLPs, has a fairly conserved sequence (Konishi and Yanagisawa, 2013; Marchive et al., 2013; Chardin et al., 2014; Liu et al., 2017). Additionally, the active RWPYRK motif of NLPs can bind to nitrate-responsive *cis*-elements (NREs), thereby promoting the expression of nitrate-inducible genes (Konishi and Yanagisawa, 2011, 2013). Indeed, post-translationally activated NLP7 is important in the PNR pathway, both under nitrogen-sufficient and nitrogen-deficient conditions. In *NLP7*-overexpressing plants, genes involved in nitrate transport (*NRT1.1*, *NRT2.1*, *NRT2.2*), nitrogen assimilation (*GS1*, *NIA1*, *NIA2*, *NIR1*), and nitrogen/auxin signal transduction (*ANR1*, *AFB3*) are upregulated to improve NUE and promote the development of a better root system (Yu et al., 2016).

#### ANR1

ANR1, an *Arabidopsis* MADS-box TF initially isolated in a screen for nitrate-inducible genes in roots, acts downstream of NRT1.1 and Ca^2+^ signaling and regulates the preferential proliferation of LRs in nitrate-rich regions in response to localized nitrate application (Zhang and Forde, 1998). Similar to the LR growth phenotype of transgenic lines that underexpress *ANR1*, LR proliferation in *AtNRT1.1* mutants is impaired in nitrate-rich zones and *ANR1* transcript levels are markedly decreased in the apical region of LRs (Remans et al., 2006). Interestingly, chromatin immunoprecipitation chip assays demonstrated that NLP7 binds to the *ANR1* locus, although there is no evidence that ANR1 functions downstream of NLP7 (Marchive et al., 2013). The detailed mechanism underlying ANR1-mediated LR initiation and growth was confirmed through the heterologous expression of *Chrysanthemum morifolium ANR1* (*CmANR1*, a homolog of *Arabidopsis AtANR1*). *CmANR1* promotes auxin accumulation in LRs and LR primordia by increasing the polar transport of auxin and its biosynthesis in roots (Sun et al., 2018). In this process, ANR1 forms homodimers and/or heterodimers with another homolog (AGL21) *via* its C-terminal region.

Local nitrate supply does not stimulate LR proliferation in *axr4* mutants, like that seen in *aux1* mutants (Zhang et al., 1999). Additionally, the asymmetric (polar) localization of AUX1 in the plasma membrane of leaf protophloem and epidermal cells was shown to be impaired in *axr4* mutants, which was not unexpected given that AXR4 mediates the localization and migration of AUX1 from the ER to the plasma membrane (Dharmasiri et al., 2006; Hobbie and Estelle, 2010). Analysis of auxin response reporter (*IAA2::GUS*) expression revealed that lateral IAA reflux and rootward/shootward auxin transport are altered in *aux1* mutant, mainly due to a reduction in the amount of the root-derived auxin that was transferred to the root apex (Swarup et al., 2001; Marchant et al., 2002). A model of a three-layer, carrier-mediated lateral auxin gradient of the elongation zone proposed by Swarup et al. (2005): PIN2 in the epidermis and cortex; weak PIN1 expression in the epidermis, cortex and endodermis; and AUX1 in the epidermis including the entire elongation zone, illustrates the role of different levels of shootward auxin flow regulated by three-layer transporters in lateral auxin gradient formation. In addition, root gravitropism is dependent on epidermal AUX1 expression, as evidenced by the fact that expanding cells migrate through the elongation zone (Swarup et al., 2005). Consequently, in the *axr4* mutant, cell division of pericycle cells and the differential expansion of epidermal cells resulting from AUX1-dependent auxin gradient generation are impaired, which inhibits LR initiation and gravitropic curvature (Marchant et al., 2002; Swarup et al., 2005). Moreover, experiments using *axr4* mutants and *ANR1* antisense lines confirmed that ANR1 acts upstream of AXR4 in local nitrate responses (Zhang et al., 1999), thus providing further evidence that ANR1-mediated LR regulation is related to PAT.

#### NAC4

NAC4, a member of the plant-specific NAM/ATAF/CUC (NAC) family of TFs, and OBP4, belonging to the DOF (DNA-binding with one finger) TF family, constitute a nitrate response regulatory module controlled by AFB3 that acts specifically in pericyclic cells to control LR initiation in response to changes in nitrate availability (Vidal et al., 2013; Asim et al., 2020). Although the function of NAC4 in plant development is not clear at present, it is known that NAC1 and NAC2 in proteins homologous to NAC4 play an intermediary role in auxin-induced LR development in *Arabidopsis*. *NAC1*, the first *NAC* gene shown to be involved in root development, is active in the apical region (meristem and elongation zone) and the LR initiation region. In pericycle cells, NAC1 protein acts downstream of the auxin signaling cascade and transmits auxin signals by activating expression from the promoters of auxin response genes such as *DBP* and *AIR3* (Xie et al., 2000). *AtNAC2* was identified by comparing the cDNA libraries of transgenic *Arabidopsis* plants overexpressing *NTHK1* (tobacco ethylene receptor gene) under salt stress with those of WT plants under similar stress. *AtNAC2* promotes LR formation, and its expression is dependent on the ethylene and auxin signaling pathways (He et al., 2005). Analogous to *NAC1/2*, AUX/IAA–ARF module-mediated auxin signaling is required for the induction of *NAC4* by nitrate. The responses of *NAC4* and *OBP4* to nitrate are altered in both *IAA14* gain-of-function and deletion mutants, suggesting that *NAC4* and *OBP4* are specifically regulated and act downstream of the AUX/IAA pathway, which affects LRP initiation in pericycle cells (Vidal et al., 2013).

A recent study showed that although OBP4 induces pericycle cell division in plants grown in auxin-containing medium, plants overexpressing OBP4 without auxin supply do not undergo cell division and have fewer LRP initials shorter cells (Ramirez-Parra et al., 2017). This is inconsistent with the widely accepted theory of “compensated cell enlargement,” which holds that a reduction in cell number can be compensated for by increasing the cell size, and vice versa (Tsukaya, 2003). OBP4 is suggested to be a negative regulator of cell cycle progression and cell expansion. Using RT-qPCR analysis, Xu et al. (2016) found that genes encoding cell wall expansion factors, such as xyloglucan endoglycosylation-related genes (*XTH3*, *XTH9*, *XTH17*); cell cycle-related genes, such as cyclin genes (*CYCA2;1, CYCA2;3*, *CYCB1;1*, *CYCB2;1*, *CYCB2;3*, and *CYCB3;1*); and CDK encoding genes (*CDKB1;1*, *CDKB1;2*, and *CDKB2;1*), were significantly inhibited in plants constitutively overexpressing OBP4. However, the predicted DOF binding sites ([A/T]AAAG and CTTT[A/T]) were only detected in the promoter regions of *CYCB1;1*, *CDKB1;1*, *XTH9*, and *XTH17* (Xu et al., 2016). These *XTH* genes encode xyloglucan endotransglucosylases/hydrolases that catalyze the cleavage of xyloglucan chains and molecular grafting between xyloglucans, resulting in wall loosening and rearrangements for cell expansion (Hyodo et al., 2003). Among the *XTH* genes, *XTH9* reduces LR density in response to increasing concentrations of nitrate, and this nitrate regulation of *XTH9* is impaired in the background of *IAA14-1* and *afb-3* mutation (Xu and Cai, 2019). And *XTH9* is also negatively regulated by *OBP4*. Combined, these observations suggest that the *XTH9-OBP4* regulatory module acts downstream of the AFB3-IAA14 module and elaborately controls LRP initiation in response to environmental nitrate availability.

#### CLEs

Under nitrogen deficiency, besides the local inhibition of LR initiation due predominantly to the transport function of NRT1.1, the whole plant translates the nitrogen-deficient nutritional status into a morphological response by suppressing the emergence and growth of LRPs. Araya et al. (2014b) identified several nitrogen-responsive CLAVATA (CLV)/ENDOSPERM SURROUNDING REGION (ESR)-related peptides (CLE1 to 7) that are induced by systematic nitrogen deficiency. CLV3 in the shoot apical meristem binds to the LRR receptor-like kinase (LRR-RLK) CLV1 located in the plasma membrane, thereby blocking WUSCHEL (WUS) and FANTASTIC FOUR 2 (*FAF2*) gene expression through calcium waves, which limits stem cell proliferation (Chou et al., 2016). These CLE peptides also form signal modules with CLV1 to prevent the expansion of the LR system into low-nitrogen environments (Araya et al., 2014a). Furthermore, there is an overaccumulation of *CLE2*, *−3*, *−4*, and *−7* transcripts in *clv1*–*4* and *clv1*–*15* mutants, suggesting that CLV1 feedback regulates CLE mRNA levels in the root apical meristem, as observed for CLV3 (Araya et al., 2014a).

#### TCP20

TCP20 belongs to the plant-specific, TEOSINTE BRANCHED1/CYCLOIDEA/PROLIFERATING CELL FACTOR1 (TCP) family of TFs and is involved in the control of cell division, expansion, and differentiation during different stages of plant morphogenesis (Guan et al., 2014). TCP20 recognizes and binds to the GCCCR (R=A or G) motif found in the promoters of several genes, including the mitotic cyclin (G2 to M phase) gene *CYCB1;1*. The transcriptional levels of *CYCB1;1* are tightly linked to the mitotic activity of apical meristem cells, which explains the functional redundancy of TCP20 in organ formation and development (Himanen et al., 2003; Li et al., 2005; Herve et al., 2009). NLP7-binding motifs have also been identified in the promoter region of *CYCB1;1*. Interestingly, although TCP20 nuclear localization is independent of nitrate availability, NLP7/TCP20 homo- and heterodimers can be retained in the nucleus under conditions of nitrogen deprivation. This not only suggests that TCP and NLP7 act together to control cycle cell-related gene expression but also implies that the activity of these homo−/heterodimers in the nucleus represents a stress response of plants induced by nitrogen starvation (Guan et al., 2017). Indeed, the transcriptional complexes bind to *CYCB1;1*, thereby preventing premature cell cycle exit, as well as to nitrate-responsive genes (*NRT1.1*, *NRT2.1*, and *NIA1*), which facilitates nitrate transport, assimilation, and signaling (Guan, 2017; Fredes et al., 2019). This suggests that TCP20 plays a key role in the systemic nitrate signaling pathway that controls root cell division. Furthermore, although PR and LR development progresses normally on homogeneous media, insertional mutations in *TCP20* strongly impair the preferential growth of LRs in high-nitrate zones (root foraging). Guan et al. (2014) showed that TCP20 regulates root foraging for nitrate by upregulating the transcriptional levels of *NRT2.1* and *NIA1* in the systemic nitrate signaling pathway.

## Conclusion

To date, the molecular mechanisms underlying the effects of nutrients and plant hormones on the RSA have mostly been elucidated through studies conducted on *Arabidopsis*. With the increase in research on nitrate and auxin, knowledge regarding how these two signals influence RSA at the genetic level has increased dramatically. It is increasingly evident that nitrate and auxin signaling pathways are not mutually exclusive; instead, extensive nitrate-associated signaling cascades are integrated into the internal auxin pathways. Auxin dominates almost all aspects of plant growth, especially root development. Roots can adapt to the fluctuating soil environment by constantly adjusting auxin distribution, thereby maximizing the exploration of external resources to ensure overall plant development.

As shown in Figure 1, the root system can obtain information about the uneven distribution of nitrate, and two sets of strategies are employed to adjust the distribution of auxin to maximize NUE. However, in a fluctuating soil environment, the roots system in low-nitrate areas mainly overcome the internal nitrogen deficiency. The shootward auxin transport regulated by NRT1.1 greatly reduces the auxin concentration in pericycle cells and prevents roots from further expanding into nitrate-poorer regions, which would aggravate internal N deficiency. In areas with high-nitrate content, plants focus on promoting LR growth to maximize nitrate acquisition, which limits the allocation of auxin from root to shoot. Furthermore, the entire root system shares the nitrogen deficiency signals, while TCP20-NLP7 dimers enhance N metabolism in low-nitrate areas and enhance root foraging in high-nitrate zones. In these processes, both local and systemic nitrate signals are integrated into the auxin response to regulate RSA.

The sustainable improvement of NUE is key to overcoming the limitations of agricultural productivity globally. Consequently, understanding how nitrate and hormone signaling are regulated and how they interact are at the core of addressing these challenges. Although current knowledge of the crosstalk between nitrate and auxin signaling is confined to the local and systemic pathway levels, it nonetheless contributes to our understanding of the regulatory network underlying nitrate signaling and its association with auxin and other phytohormone signaling pathways that are key for developing new biotechnology-based strategies and achieving sustainable agriculture. The emergence of tools such as cell type-specific or single-cell genomics and proteomics following on from the sequencing of the *Arabidopsis* genome has greatly improved our understanding of how plants integrate internal and external information.

## Author Contributions

Q-QH, J-QS, and W-ML: manuscript preparation. G-ZW: manuscript review and editing. All authors contributed to the article and approved the submitted version.

## Funding

This research was financially supported by the Basic Research Project of Science and Technology, Department of Sichuan Province (grant no. 2021YJ0110).

## Conflict of Interest

The authors declare that the research was conducted in the absence of any commercial or financial relationships that could be construed as a potential conflict of interest.

## Publisher’s Note

All claims expressed in this article are solely those of the authors and do not necessarily represent those of their affiliated organizations, or those of the publisher, the editors and the reviewers. Any product that may be evaluated in this article, or claim that may be made by its manufacturer, is not guaranteed or endorsed by the publisher.

## References

[ref1] Adamowski M. Friml J. (2015). PIN-dependent auxin transport: action, regulation, and evolution. Plant Cell 27, 20–32. doi: 10.1105/tpc.114.134874, PMID: 25604445 PMC4330589

[ref2] Aida M. Beis D. Heidstra R. Willemsen V. Blilou I. Galinha C.. (2004). The PLETHORA genes mediate patterning of the *Arabidopsis* root stem cell niche. Cell 119, 109–120. doi: 10.1016/j.cell.2004.09.018, PMID: 15454085

[ref3] Alvarez J. M. Vidal E. A. Gutierrez R. A. (2012). Integration of local and systemic signaling pathways for plant N responses. Curr. Opin. Plant Biol. 15, 185–191. doi: 10.1016/j.pbi.2012.03.009, PMID: 22480431

[ref4] Arase F. Nishitani H. Egusa M. Nishimoto N. Sakurai S. Sakamoto N.. (2012). IAA8 involved in lateral root formation interacts with the TIR1 auxin receptor and ARF transcription factors in *Arabidopsis*. PLoS One 7:e43414. doi: 10.1371/journal.pone.0043414, PMID: 22912871 PMC3422273

[ref5] Araya T. Miyamoto M. Wibowo J. Suzuki A. Kojima S. Tsuchiya Y. N.. (2014a). CLE-CLAVATA1 peptide-receptor signaling module regulates the expansion of plant root systems in a nitrogen-dependent manner. Proc. Natl. Acad. Sci. U. S. A. 111, 2029–2034. doi: 10.1073/pnas.1319953111, PMID: 24449877 PMC3918772

[ref6] Araya T. Von Wiren N. Takahashi H. (2014b). CLE peptides regulate lateral root development in response to nitrogen nutritional status of plants. Plant Signal. Behav. 9:e29302. doi: 10.4161/psb.29302, PMID: 25763500 PMC4203639

[ref7] Asim M. Ullah Z. Oluwaseun A. Wang Q. Liu H. (2020). Signalling overlaps between nitrate and auxin in regulation of the root system architecture: insights from the *Arabidopsis thaliana*. Int. J. Mol. Sci. 21:2880. doi: 10.3390/ijms21082880, PMID: 32326090 PMC7215989

[ref8] Bellini C. Pacurar D. I. Perrone I. (2014). Adventitious roots and lateral roots: similarities and differences. Annu. Rev. Plant Biol. 65, 639–666. doi: 10.1146/annurev-arplant-050213-035645, PMID: 24555710

[ref9] Benfey P. N. Scheres B. (2000). Root development. Curr. Biol. 10, R813–R815. doi: 10.1016/S0960-9822(00)00814-9, PMID: 11102819

[ref10] Berridge M. J. (2016). The inositol trisphosphate/calcium signaling pathway in health and disease. Physiol. Rev. 96, 1261–1296. doi: 10.1152/physrev.00006.2016, PMID: 27512009

[ref11] Bhalerao R. P. Eklöf J. Ljung K. Marchant A. Bennett M. Sandberg G. (2002). Shoot-derived auxin is essential for early lateral root emergence in *Arabidopsis* seedlings. Plant J. 29, 325–332. doi: 10.1046/j.0960-7412.2001.01217.x, PMID: 11844109

[ref12] Bisseling T. Scheres B. (2014). Nutrient computation for root architecture: perspective. Science 346, 300–301. doi: 10.1126/science.1260942, PMID: 25324371

[ref13] Brown J. C. Jones A. M. (1994). Mapping the auxin-binding site of auxin-binding protein 1. J. Biol. Chem. 269, 21136–21140. doi: 10.1016/S0021-9258(17)31940-3 8063734

[ref14] Brumos J. Robles L. M. Yun J. Vu T. C. Jackson S. Alonso J. M.. (2018). Local auxin biosynthesis is a key regulator of plant development. Dev. Cell 47, 306–318.e5. doi: 10.1016/j.devcel.2018.09.022, PMID: 30415657

[ref15] Cai Z. Wang Y. Zhu L. Tian Y. Chen L. Sun Z.. (2017). GmTIR1/GmAFB3-based auxin perception regulated by miR393 modulates soybean nodulation. New Phytol. 215, 672–686. doi: 10.1111/nph.14632, PMID: 28598036

[ref16] Cao X. Yang H. Shang C. Ma S. Liu L. Cheng J. (2019). The roles of auxin biosynthesis YUCCA gene family in plants. Int. J. Mol. Sci. 20:6343. doi: 10.3390/ijms20246343, PMID: 31888214 PMC6941117

[ref17] Casimiro I. Beeckman T. Graham N. Bhalerao R. Zhang H. Casero P.. (2003). Dissecting *Arabidopsis* lateral root development. Trends Plant Sci. 8, 165–171. doi: 10.1016/S1360-1385(03)00051-7, PMID: 12711228

[ref18] Castaings L. Camargo A. Pocholle D. Gaudon V. Texier Y. Boutet-Mercey S.. (2009). The nodule inception-like protein 7 modulates nitrate sensing and metabolism in *Arabidopsis*. Plant J. 57, 426–435. doi: 10.1111/j.1365-313X.2008.03695.x, PMID: 18826430

[ref19] Cerezo M. Tillard P. Filleur S. Muños S. Daniel-Vedele F. Gojon A. (2001). Major alterations of the regulation of root NO(3)(−) uptake are associated with the mutation of Nrt2.1 and Nrt2.2 genes in *Arabidopsis*. Plant Physiol. 127, 262–271. doi: 10.1104/pp.127.1.262, PMID: 11553754 PMC117982

[ref20] Chardin C. Girin T. Roudier F. Meyer C. Krapp A. (2014). The plant RWP-RK transcription factors: key regulators of nitrogen responses and of gametophyte development. J. Exp. Bot. 65, 5577–5587. doi: 10.1093/jxb/eru261, PMID: 24987011

[ref21] Chen Z. H. Bao M. L. Sun Y. Z. Yang Y. J. Xu X. H. Wang J. H.. (2011). Regulation of auxin response by miR393-targeted transport inhibitor response protein 1 is involved in normal development in *Arabidopsis*. Plant Mol. Biol. 77, 619–629. doi: 10.1007/s11103-011-9838-1, PMID: 22042293

[ref22] Chen Q. Dai X. De-Paoli H. Cheng Y. Takebayashi Y. Kasahara H.. (2014). Auxin overproduction in shoots cannot rescue auxin deficiencies in *Arabidopsis* roots. Plant Cell Physiol. 55, 1072–1079. doi: 10.1093/pcp/pcu039, PMID: 24562917 PMC4051135

[ref23] Chen Q. Liu Y. Maere S. Lee E. Van Isterdael G. Xie Z.. (2015). A coherent transcriptional feed-forward motif model for mediating auxin-sensitive PIN3 expression during lateral root development. Nat. Commun. 6:8821. doi: 10.1038/ncomms9821, PMID: 26578065 PMC4673502

[ref24] Chen X. Naramoto S. Robert S. Tejos R. Lofke C. Lin D.. (2012). ABP1 and ROP6 GTPase signaling regulate clathrin-mediated endocytosis in *Arabidopsis* roots. Curr. Biol. 22, 1326–1332. doi: 10.1016/j.cub.2012.05.020, PMID: 22683261

[ref25] Cho M. Cho H. (2013). The function of ABCB transporters in auxin transport. Plant Signal. Behav. 8:e22990. doi: 10.4161/psb.22990, PMID: 23221777 PMC3656995

[ref490] Chou H. Zhu Y. Ma Y. Berkowitz G. A. (2016). The CLAVATA signaling pathway mediating stem cell fate in shoot meristems requires Ca(^2+^) as a secondary cytosolic messenger. Plant. J. 85, 494–506. doi: 10.1111/tpj.13123, PMID: 26756833

[ref26] Ciechanover A. (1998). The ubiquitin-proteasome pathway: on protein death and cell life. EMBO J. 17, 7151–7160. doi: 10.1093/emboj/17.24.7151, PMID: 9857172 PMC1171061

[ref27] Clowes F. A. L. (2010). Nucleic acids in root apical meristems of *Zea*. New Phytol. 55, 29–34. doi: 10.1111/j.1469-8137.1956.tb05264.x

[ref28] Crawford N. M. (1995). Nitrate: nutrient and signal for plant growth. Plant Cell 7, 859–868. doi: 10.1105/tpc.7.7.859, PMID: 7640524 PMC160877

[ref29] Crawford N. M. Glass A. D. M. (1998). Molecular and physiological aspects of nitrate uptake in plants. Trends Plant Sci. 3, 389–395. doi: 10.1016/S1360-1385(98)01311-9

[ref30] Dastidar M. G. Scarpa A. Magele I. Ruiz-Duarte P. Von Born P. Bald L.. (2019). ARF5/MONOPTEROS directly regulates miR390 expression in the *Arabidopsis thaliana* primary root meristem. Plant Direct 3:e00116. doi: 10.1002/pld3.116, PMID: 31245759 PMC6508847

[ref31] De Smet I. Lau S. Voss U. Vanneste S. Benjamins R. Rademacher E. H.. (2010). Bimodular auxin response controls organogenesis in *Arabidopsis*. Proc. Natl. Acad. Sci. U. S. A. 107, 2705–2710. doi: 10.1073/pnas.0915001107, PMID: 20133796 PMC2823897

[ref32] Dharmasiri N. Dharmasiri S. Estelle M. (2005a). The F-box protein TIR1 is an auxin receptor. Nature 435, 441–445. doi: 10.1038/nature03543, PMID: 15917797

[ref33] Dharmasiri N. Dharmasiri S. Weijers D. Lechner E. Yamada M. Hobbie L.. (2005b). Plant development is regulated by a family of auxin receptor F box proteins. Dev. Cell 9, 109–119. doi: 10.1016/j.devcel.2005.05.014, PMID: 15992545

[ref34] Dharmasiri S. Swarup R. Mockaitis K. Dharmasiri N. Singh S. K. Kowalchyk M.. (2006). AXR4 is required for localization of the auxin influx facilitator AUX1. Science 312, 1218–1220. doi: 10.1126/science.1122847, PMID: 16690816

[ref35] Ding Z. Friml J. (2010). Auxin regulates distal stem cell differentiation in *Arabidopsis* roots. Proc. Natl. Acad. Sci. U. S. A. 107, 12046–12051. doi: 10.1073/pnas.1000672107, PMID: 20543136 PMC2900669

[ref36] Dolan L. Janmaat K. Willemsen V. Linstead P. Poethig S. Roberts K.. (1993). Cellular organisation of the *Arabidopsis thaliana* root. Development 119, 71–84. doi: 10.1242/dev.119.1.71, PMID: 8275865

[ref37] Dubrovsky J. G. Rost T. L. Colon-Carmona A. Doerner P. (2001). Early primordium morphogenesis during lateral root initiation in *Arabidopsis thaliana*. Planta 214, 30–36. doi: 10.1007/s004250100598, PMID: 11762168

[ref38] Feng Z. Sun X. Wang G. Liu H. Zhu J. (2012). LBD29 regulates the cell cycle progression in response to auxin during lateral root formation in *Arabidopsis thaliana*. Ann. Bot. 110, 1–10. doi: 10.1093/aob/mcs019, PMID: 22334497 PMC3380585

[ref39] Forde B. G. (2002). Local and long-range signaling pathways regulating plant responses to nitrate. Annu. Rev. Plant Biol. 53, 203–224. doi: 10.1146/annurev.arplant.53.100301.135256, PMID: 12221973

[ref40] Fornerod M. Ohno M. Yoshida M. Mattaj I. W. (1997). CRM1 is an export receptor for leucine-rich nuclear export signals. Cell 90, 1051–1060. doi: 10.1016/S0092-8674(00)80371-2, PMID: 9323133

[ref41] Fredes I. Moreno S. Diaz F. P. Gutierrez R. A. (2019). Nitrate signaling and the control of *Arabidopsis* growth and development. Curr. Opin. Plant Biol. 47, 112–118. doi: 10.1016/j.pbi.2018.10.004, PMID: 30496968

[ref42] Galinha C. Hofhuis H. Luijten M. Willemsen V. Blilou I. Heidstra R.. (2007). PLETHORA proteins as dose-dependent master regulators of *Arabidopsis* root development. Nature 449, 1053–1057. doi: 10.1038/nature06206, PMID: 17960244

[ref43] Gälweiler L. Guan C. Müller A. Wisman E. Mendgen K. Yephremov A.. (1998). Regulation of polar auxin transport by AtPIN1 in *Arabidopsis* vascular tissue. Science 282, 2226–2230. doi: 10.1126/science.282.5397.2226, PMID: 9856939

[ref44] Gao Y. Zhang Y. Zhang D. Dai X. Estelle M. Zhao Y. (2015). Auxin binding protein 1 (ABP1) is not required for either auxin signaling or *Arabidopsis* development. Proc. Natl. Acad. Sci. U. S. A. 112, 2275–2280. doi: 10.1073/pnas.1500365112, PMID: 25646447 PMC4343106

[ref45] Geisler M. Murphy A. S. (2006). The ABC of auxin transport: the role of p-glycoproteins in plant development. FEBS Lett. 580, 1094–1102. doi: 10.1016/j.febslet.2005.11.054, PMID: 16359667

[ref46] Gifford M. L. Dean A. Gutierrez R. A. Coruzzi G. M. Birnbaum K. D. (2008). Cell-specific nitrogen responses mediate developmental plasticity. Proc. Natl. Acad. Sci. U. S. A. 105, 803–808. doi: 10.1073/pnas.0709559105, PMID: 18180456 PMC2206617

[ref47] Goh T. Joi S. Mimura T. Fukaki H. (2012a). The establishment of asymmetry in *Arabidopsis* lateral root founder cells is regulated by LBD16/ASL18 and related LBD/ASL proteins. Development 139, 883–893. doi: 10.1242/dev.071928, PMID: 22278921

[ref48] Goh T. Kasahara H. Mimura T. Kamiya Y. Fukaki H. (2012b). Multiple AUX/IAA-ARF modules regulate lateral root formation: the role of *Arabidopsis* SHY2/IAA3-mediated auxin signalling. Philos. Trans. R. Soc. Lond. Ser. B Biol. Sci. 367, 1461–1468. doi: 10.1098/rstb.2011.0232, PMID: 22527388 PMC3321683

[ref49] Gojon A. Krouk G. Perrine-Walker F. Laugier E. (2011). Nitrate transceptor(s) in plants. J. Exp. Bot. 62, 2299–2308. doi: 10.1093/jxb/erq419, PMID: 21239382

[ref50] Gray W. M. Kepinski S. Rouse D. Leyser O. Estelle M. (2001). Auxin regulates SCF(TIR1)-dependent degradation of AUX/IAA proteins. Nature 414, 271–276. doi: 10.1038/35104500, PMID: 11713520

[ref51] Grieneisen V. A. Scheres B. Hogeweg P. Af M. M. (2012). Morphogengineering roots: comparing mechanisms of morphogen gradient formation. BMC Syst. Biol. 6:37. doi: 10.1186/1752-0509-6-37, PMID: 22583698 PMC3681314

[ref52] Guan P. (2017). Dancing with hormones: a current perspective of nitrate signaling and regulation in *Arabidopsis*. Front. Plant Sci. 8:1697. doi: 10.3389/fpls.2017.01697, PMID: 29033968 PMC5625010

[ref53] Guan P. Ripoll J. J. Wang R. Vuong L. Bailey-Steinitz L. J. Ye D.. (2017). Interacting TCP and NLP transcription factors control plant responses to nitrate availability. Proc. Natl. Acad. Sci. U. S. A. 114, 2419–2424. doi: 10.1073/pnas.1615676114, PMID: 28202720 PMC5338533

[ref54] Guan P. Wang R. Nacry P. Breton G. Kay S. A. Pruneda-Paz J. L.. (2014). Nitrate foraging by *Arabidopsis* roots is mediated by the transcription factor TCP20 through the systemic signaling pathway. Proc. Natl. Acad. Sci. U. S. A. 111, 15267–15272. doi: 10.1073/pnas.1411375111, PMID: 25288754 PMC4210337

[ref55] Guo F. Q. Wang R. Crawford N. M. (2002). The *Arabidopsis* dual-affinity nitrate transporter gene AtNRT1.1 (CHL1) is regulated by auxin in both shoots and roots. J. Exp. Bot. 53, 835–844. doi: 10.1093/jexbot/53.370.835, PMID: 11912226

[ref56] Gutierrez L. Bussell J. D. Pacurar D. I. Schwambach J. Pacurar M. Bellini C. (2009). Phenotypic plasticity of adventitious rooting in *Arabidopsis* is controlled by complex regulation of AUXIN RESPONSE FACTOR transcripts and microRNA abundance. Plant Cell 21, 3119–3132. doi: 10.1105/tpc.108.064758, PMID: 19820192 PMC2782293

[ref57] Gutierrez L. Mongelard G. Flokova K. Pacurar D. I. Novak O. Staswick P.. (2012). Auxin controls *Arabidopsis* adventitious root initiation by regulating jasmonic acid homeostasis. Plant Cell 24, 2515–2527. doi: 10.1105/tpc.112.099119, PMID: 22730403 PMC3406919

[ref58] Haissig B. E. (1974). Origins of adventitious roots. N. Z. J. For. Sci. 4, 299–310.

[ref59] Hamann T. Benkova E. Baurle I. Kientz M. Jurgens G. (2002). The *Arabidopsis* BODENLOS gene encodes an auxin response protein inhibiting MONOPTEROS-mediated embryo patterning. Genes Dev. 16, 1610–1615. doi: 10.1101/gad.229402, PMID: 12101120 PMC186366

[ref60] Haruta M. Gray W. M. Sussman M. R. (2015). Regulation of the plasma membrane proton pump (H(+)-ATPase) by phosphorylation. Curr. Opin. Plant Biol. 28, 68–75. doi: 10.1016/j.pbi.2015.09.005, PMID: 26476298 PMC4679459

[ref61] He X. J. Mu R. L. Cao W. H. Zhang Z. G. Zhang J. S. Chen S. Y. (2005). AtNAC2, a transcription factor downstream of ethylene and auxin signaling pathways, is involved in salt stress response and lateral root development. Plant J. 44, 903–916. doi: 10.1111/j.1365-313X.2005.02575.x, PMID: 16359384

[ref62] Herve C. Dabos P. Bardet C. Jauneau A. Auriac M. C. Ramboer A.. (2009). In vivo interference with AtTCP20 function induces severe plant growth alterations and deregulates the expression of many genes important for development. Plant Physiol. 149, 1462–1477. doi: 10.1104/pp.108.126136, PMID: 19091878 PMC2649380

[ref63] Himanen K. Reuzeau C. Beeckman T. Melzer S. Grandjean O. Corben L.. (2003). The *Arabidopsis* locus RCB mediates upstream regulation of mitotic gene expression. Plant Physiol. 133, 1862–1872. doi: 10.1104/pp.103.027128, PMID: 14681535 PMC300739

[ref64] Ho C. H. Lin S. H. Hu H. C. Tsay Y. F. (2009). CHL1 functions as a nitrate sensor in plants. Cell 138, 1184–1194. doi: 10.1016/j.cell.2009.07.004, PMID: 19766570

[ref65] Hobbie L. Estelle M. (2010). The axr4 auxin-resistant mutants of *Arabidopsis thaliana* define a gene important for root gravitropism and lateral root initiation. Plant J. 7, 211–220. doi: 10.1046/j.1365-313X.1995.7020211.x, PMID: 7704045

[ref66] Hu H. C. Wang Y. Y. Tsay Y. F. (2009). AtCIPK8, a CBL-interacting protein kinase, regulates the low-affinity phase of the primary nitrate response. Plant J. 57, 264–278. doi: 10.1111/j.1365-313X.2008.03685.x, PMID: 18798873

[ref67] Hull A. K. Vij R. Celenza J. L. (2000). *Arabidopsis* cytochrome P450s that catalyze the first step of tryptophan-dependent indole-3-acetic acid biosynthesis. Proc. Natl. Acad. Sci. U. S. A. 97, 2379–2384. doi: 10.1073/pnas.040569997, PMID: 10681464 PMC15809

[ref68] Hyodo H. Yamakawa S. Takeda Y. Tsuduki M. Yokota A. Nishitani K.. (2003). Active gene expression of a xyloglucan endotransglucosylase/hydrolase gene, XTH9, in inflorescence apices is related to cell elongation in *Arabidopsis thaliana*. Plant Mol. Biol. 52, 473–482. doi: 10.1023/A:1023904217641, PMID: 12856951

[ref69] Jing H. Strader L. C. (2019). Interplay of auxin and cytokinin in lateral root development. Int. J. Mol. Sci. 20:486. doi: 10.3390/ijms20030486, PMID: 30678102 PMC6387363

[ref70] Jones-Rhoades M. W. Bartel D. P. Bartel B. (2006). MicroRNAS and their regulatory roles in plants. Annu. Rev. Plant Biol. 57, 19–53. doi: 10.1146/annurev.arplant.57.032905.105218, PMID: 16669754

[ref71] Kim Y. S. Min J. K. Kim D. Jung J. (2001). A soluble auxin-binding protein, ABP57. Purification with anti-bovine serum albumin antibody and characterization of its mechanistic role in the auxin effect on plant plasma membrane H+-ATPase. J. Biol. Chem. 276, 10730–10736. doi: 10.1074/jbc.M009416200, PMID: 11154693

[ref72] Koizumi K. Gallagher K. L. (2013). Identification of SHRUBBY, a SHORT-ROOT and SCARECROW interacting protein that controls root growth and radial patterning. Development 140, 1292–1300. doi: 10.1242/dev.090761, PMID: 23444357

[ref73] Konishi M. Yanagisawa S. (2011). Roles of the transcriptional regulation mediated by the nitrate-responsive cis-element in higher plants. Biochem. Biophys. Res. Commun. 411, 708–713. doi: 10.1016/j.bbrc.2011.07.008, PMID: 21777567

[ref74] Konishi M. Yanagisawa S. (2013). *Arabidopsis* NIN-like transcription factors have a central role in nitrate signalling. Nat. Commun. 4:1617. doi: 10.1038/ncomms2621, PMID: 23511481

[ref75] Korasick D. A. Enders T. A. Strader L. C. (2013). Auxin biosynthesis and storage forms. J. Exp. Bot. 64, 2541–2555. doi: 10.1093/jxb/ert080, PMID: 23580748 PMC3695655

[ref76] Krinke O. Novotna Z. Valentova O. Martinec J. (2007). Inositol trisphosphate receptor in higher plants: is it real? J. Exp. Bot. 58, 361–376. doi: 10.1093/jxb/erl220, PMID: 17150991

[ref77] Krouk G. (2017). Nitrate signalling: calcium bridges the nitrate gap. Nat. Plants 3:17095. doi: 10.1038/nplants.2017.95, PMID: 28628086

[ref78] Krouk G. Lacombe B. Bielach A. Perrine-Walker F. Malinska K. Mounier E.. (2010). Nitrate-regulated auxin transport by NRT1.1 defines a mechanism for nutrient sensing in plants. Dev. Cell 18, 927–937. doi: 10.1016/j.devcel.2010.05.008, PMID: 20627075

[ref79] Kudo N. Wolff B. Sekimoto T. Schreiner E. P. Yoneda Y. Yanagida M.. (1998). Leptomycin B inhibition of signal-mediated nuclear export by direct binding to CRM1. Exp. Cell Res. 242, 540–547. doi: 10.1006/excr.1998.4136, PMID: 9683540

[ref80] Lakehal A. Chaabouni S. Cavel E. Le Hir R. Ranjan A. Raneshan Z.. (2019). A molecular framework for the control of adventitious rooting by TIR1/AFB2-aux/IAA-dependent auxin signaling in *Arabidopsis*. Mol. Plant 12, 1499–1514. doi: 10.1016/j.molp.2019.09.001, PMID: 31520787

[ref81] Lam H. M. Coschigano K. Schultz C. Melo-Oliveira R. Tjaden G. Oliveira I.. (1995). Use of *Arabidopsis* mutants and genes to study amide amino acid biosynthesis. Plant Cell 7, 887–898. doi: 10.1105/tpc.7.7.887, PMID: 7640525 PMC160882

[ref82] Lay-Pruitt K. S. Takahashi H. (2020). Integrating N signals and root growth: the role of nitrate transceptor NRT1.1 in auxin-mediated lateral root development. J. Exp. Bot. 71, 4365–4368. doi: 10.1093/jxb/eraa243, PMID: 32710785 PMC7382374

[ref83] Lee H. W. Cho C. Pandey S. K. Park Y. Kim M. J. Kim J. (2019). LBD16 and LBD18 acting downstream of ARF7 and ARF19 are involved in adventitious root formation in *Arabidopsis*. BMC Plant Biol. 19:46. doi: 10.1186/s12870-019-1659-4, PMID: 30704405 PMC6357364

[ref84] Lee R. C. Feinbaum R. L. Ambros V. (1993). The *C. elegans* heterochronic gene lin-4 encodes small RNAs with antisense complementarity to lin-14. Cell 75, 843–854. doi: 10.1016/0092-8674(93)90529-Y, PMID: 8252621

[ref85] Lee K. Kim M.-I. Kwon Y.-J. Kim M. Kim Y.-S. Kim D. (2009). Cloning and characterization of a gene encoding ABP57, a soluble auxin-binding protein. Plant Biotechnol. Rep. 3, 293–299. doi: 10.1007/s11816-009-0101-z

[ref86] Lee R. B. Rudge K. A. (1986). Effects of nitrogen deficiency on the absorption of nitrate and ammonium by barley plants. Ann. Bot. 57, 471–486. doi: 10.1093/oxfordjournals.aob.a087129

[ref87] Lemtiri C. F. Macrobbie E. A. Webb A. A. Manison N. F. Brownlee C. Skepper J. N.. (2003). Inositol hexakisphosphate mobilizes an endomembrane store of calcium in guard cells. Proc. Natl. Acad. Sci. U. S. A. 100, 10091–10095. doi: 10.1073/pnas.1133289100, PMID: 12913129 PMC187775

[ref88] Li C. Potuschak T. Colón-Carmona A. Gutiérrez R. A. Doerner P. (2005). *Arabidopsis* TCP20 links regulation of growth and cell division control pathways. Proc. Natl. Acad. Sci. U. S. A. 102, 12978–12983. doi: 10.1073/pnas.0504039102, PMID: 16123132 PMC1200278

[ref89] Lin D. Nagawa S. Chen J. Cao L. Chen X. Xu T.. (2012). A ROP GTPase-dependent auxin signaling pathway regulates the subcellular distribution of PIN2 in *Arabidopsis* roots. Curr. Biol. 22, 1319–1325. doi: 10.1016/j.cub.2012.05.019, PMID: 22683260 PMC3407329

[ref90] Little D. Y. Rao H. Oliva S. Daniel-Vedele F. Krapp A. Malamy J. E. (2005). The putative high-affinity nitrate transporter NRT2.1 represses lateral root initiation in response to nutritional cues. Proc. Natl. Acad. Sci. U. S. A. 102, 13693–13698. doi: 10.1073/pnas.0504219102, PMID: 16157886 PMC1224627

[ref91] Liu K. H. Niu Y. Konishi M. Wu Y. Du H. Sun Chung H.. (2017). Discovery of nitrate-CPK-NLP signalling in central nutrient-growth networks. Nature 545, 311–316. doi: 10.1038/nature22077, PMID: 28489820 PMC5823009

[ref92] Liu K. H. Tsay H. Y. F. (1999). CHL1 is a dual-affinity nitrate transporter of *Arabidopsis* involved in multiple phases of nitrate uptake. Plant Cell 11, 865–874. doi: 10.1105/tpc.11.5.865, PMID: 10330471 PMC144217

[ref93] Liu K. H. Tsay Y. F. (2003). Switching between the two action modes of the dual-affinity nitrate transporter CHL1 by phosphorylation. EMBO J. 22, 1005–1013. doi: 10.1093/emboj/cdg118, PMID: 12606566 PMC150351

[ref94] Ljung K. Hull A. K. Celenza J. Yamada M. Estelle M. Normanly J.. (2005). Sites and regulation of auxin biosynthesis in *Arabidopsis* roots. Plant Cell 17, 1090–1104. doi: 10.1105/tpc.104.029272, PMID: 15772288 PMC1087988

[ref95] Lopez-Bucio J. (2003). The role of nutrient availability in regulating root architecture. Curr. Opin. Plant Biol. 6, 280–287. doi: 10.1016/S1369-5266(03)00035-9, PMID: 12753979

[ref96] Lu Y. Feng Z. Liu X. Bian L. Xie H. Zhang C.. (2018). MiR393 and miR390 synergistically regulate lateral root growth in rice under different conditions. BMC Plant Biol. 18:261. doi: 10.1186/s12870-018-1488-x, PMID: 30373525 PMC6206659

[ref97] Ma W. Li J. Qu B. He X. Zhao X. Li B.. (2014). Auxin biosynthetic gene TAR2 is involved in low nitrogen-mediated reprogramming of root architecture in *Arabidopsis*. Plant J. 78, 70–79. doi: 10.1111/tpj.12448, PMID: 24460551

[ref98] Maghiaoui A. Bouguyon E. Cuesta C. Perrine-Walker F. Alcon C. Krouk G.. (2020). The *Arabidopsis* NRT1.1 transceptor coordinately controls auxin biosynthesis and transport to regulate root branching in response to nitrate. J. Exp. Bot. 71, 4480–4494. doi: 10.1093/jxb/eraa242, PMID: 32428238

[ref99] Mano Y. Nemoto K. (2012). The pathway of auxin biosynthesis in plants. J. Exp. Bot. 63, 2853–2872. doi: 10.1093/jxb/ers091, PMID: 22447967

[ref100] Maraschin Fdos S. Memelink J. Offringa R. (2009). Auxin-induced, SCF(TIR1)-mediated poly-ubiquitination marks AUX/IAA proteins for degradation. Plant J. 59, 100–109. doi: 10.1111/j.1365-313X.2009.03854.x, PMID: 19309453

[ref101] Marchant A. Bhalerao R. Casimiro I. Eklöf J. Casero P. J. Bennett M.. (2002). AUX1 promotes lateral root formation by facilitating indole-3-acetic acid distribution between sink and source tissues in the *Arabidopsis* seedling. Plant Cell 14, 589–597. doi: 10.1105/tpc.010354, PMID: 11910006 PMC150581

[ref102] Marchive C. Roudier F. Castaings L. Brehaut V. Blondet E. Colot V.. (2013). Nuclear retention of the transcription factor NLP7 orchestrates the early response to nitrate in plants. Nat. Commun. 4:1713. doi: 10.1038/ncomms2650, PMID: 23591880

[ref103] Marhavy P. Vanstraelen M. De Rybel B. Zhaojun D. Bennett M. J. Beeckman T.. (2014). Auxin reflux between the endodermis and pericycle promotes lateral root initiation. EMBO J. 32, 149–158. doi: 10.1038/emboj.2012.303, PMID: 23178590 PMC3545298

[ref104] Marin E. Jouannet V. Herz A. Lokerse A. S. Weijers D. Vaucheret H.. (2010). miR390, *Arabidopsis* TAS3 tasiRNAs, and their AUXIN RESPONSE FACTOR targets define an autoregulatory network quantitatively regulating lateral root growth. Plant Cell 22, 1104–1117. doi: 10.1105/tpc.109.072553, PMID: 20363771 PMC2879756

[ref105] Medici A. Krouk G. (2014). The primary nitrate response: a multifaceted signalling pathway. J. Exp. Bot. 65, 5567–5576. doi: 10.1093/jxb/eru245, PMID: 24942915

[ref106] Mikkelsen M. D. Naur P. Halkier B. A. (2004). *Arabidopsis* mutants in the C-S lyase of glucosinolate biosynthesis establish a critical role for indole-3-acetaldoxime in auxin homeostasis. Plant J. 37, 770–777. doi: 10.1111/j.1365-313X.2004.02002.x, PMID: 14871316

[ref107] Mlodzinska E. Klobus G. Christensen M. D. Fuglsang A. T. (2015). The plasma membrane H(+)-ATPase AHA2 contributes to the root architecture in response to different nitrogen supply. Physiol. Plant. 154, 270–282. doi: 10.1111/ppl.12305, PMID: 25382626

[ref108] Mounier E. Pervent M. Ljung K. Gojon A. Nacry P. (2014). Auxin-mediated nitrate signalling by NRT1.1 participates in the adaptive response of *Arabidopsis* root architecture to the spatial heterogeneity of nitrate availability. Plant Cell Environ. 37, 162–174. doi: 10.1111/pce.12143, PMID: 23731054

[ref109] Nafisi M. Goregaoker S. Botanga C. J. Glawischnig E. Olsen C. E. Halkier B. A.. (2007). *Arabidopsis* cytochrome P450 monooxygenase 71A13 catalyzes the conversion of indole-3-acetaldoxime in camalexin synthesis. Plant Cell 19, 2039–2052. doi: 10.1105/tpc.107.051383, PMID: 17573535 PMC1955726

[ref110] Nibau C. Gibbs D. J. Coates J. C. (2008). Branching out in new directions: the control of root architecture by lateral root formation. New Phytol. 179, 595–614. doi: 10.1111/j.1469-8137.2008.02472.x, PMID: 18452506

[ref111] Ohkubo Y. Tanaka M. Tabata R. Ogawa-Ohnishi M. Matsubayashi Y. (2017). Shoot-to-root mobile polypeptides involved in systemic regulation of nitrogen acquisition. Nat. Plants 3:17029. doi: 10.1038/nplants.2017.29, PMID: 28319056

[ref112] Okushima Y. Fukaki H. Onoda M. Theologis A. Tasaka M. (2007). ARF7 and ARF19 regulate lateral root formation via direct activation of LBD/ASL genes in *Arabidopsis*. Plant Cell 19, 118–130. doi: 10.1105/tpc.106.047761, PMID: 17259263 PMC1820965

[ref113] Olatunji D. Geelen D. Verstraeten I. (2017). Control of endogenous auxin levels in plant root development. Int. J. Mol. Sci. 18:2587. doi: 10.3390/ijms18122587, PMID: 29194427 PMC5751190

[ref114] Parry G. Calderon-Villalobos L. I. Prigge M. Peret B. Dharmasiri S. Itoh H.. (2009). Complex regulation of the TIR1/AFB family of auxin receptors. Proc. Natl. Acad. Sci. U. S. A. 106, 22540–22545. doi: 10.1073/pnas.0911967106, PMID: 20018756 PMC2799741

[ref115] Peret B. Swarup K. Ferguson A. Seth M. Yang Y. Dhondt S.. (2012). AUX/LAX genes encode a family of auxin influx transporters that perform distinct functions during *Arabidopsis* development. Plant Cell 24, 2874–2885. doi: 10.1105/tpc.112.097766, PMID: 22773749 PMC3426120

[ref116] Petrasek J. Friml J. (2009). Auxin transport routes in plant development. Development 136, 2675–2688. doi: 10.1242/dev.030353, PMID: 19633168

[ref117] Pollmann S. Duchting P. Weiler E. W. (2009). Tryptophan-dependent indole-3-acetic acid biosynthesis by ‘IAA-synthase’ proceeds via indole-3-acetamide. Phytochemistry 70, 523–531. doi: 10.1016/j.phytochem.2009.01.021, PMID: 19268331

[ref118] Porco S. Larrieu A. Du Y. Gaudinier A. Goh T. Swarup K.. (2016). Lateral root emergence in *Arabidopsis* is dependent on transcription factor LBD29 regulation of auxin influx carrier LAX3. Development 143, 3340–3349. doi: 10.1242/dev.136283, PMID: 27578783

[ref119] Ramirez-Parra E. Perianez-Rodriguez J. Navarro-Neila S. Gude I. Moreno-Risueno M. A. Del Pozo J. C. (2017). The transcription factor OBP4 controls root growth and promotes callus formation. New Phytol. 213, 1787–1801. doi: 10.1111/nph.14315, PMID: 27859363

[ref120] Remans T. Nacry P. Pervent M. Filleur S. Diatloff E. Mounier E.. (2006). The *Arabidopsis* NRT1.1 transporter participates in the signaling pathway triggering root colonization of nitrate-rich patches. Proc. Natl. Acad. Sci. U. S. A. 103, 19206–19211. doi: 10.1073/pnas.0605275103, PMID: 17148611 PMC1748200

[ref121] Riveras E. Alvarez J. M. Vidal E. A. Oses C. Vega A. Gutierrez R. A. (2015). The calcium ion is a second messenger in the nitrate signaling pathway of *Arabidopsis*. Plant Physiol. 169, 1397–1404. doi: 10.1104/pp.15.00961, PMID: 26304850 PMC4587466

[ref122] Robert S. Kleine-Vehn J. Barbez E. Sauer M. Paciorek T. Baster P.. (2010). ABP1 mediates auxin inhibition of clathrin-dependent endocytosis in *Arabidopsis*. Cell 143, 111–121. doi: 10.1016/j.cell.2010.09.027, PMID: 20887896 PMC3503507

[ref123] Scheres B. Wolkenfelt H. Willemsen V. Terlouw M. Lawson E. Dean C.. (1994). Embryonic origin of the *Arabidopsis* primary root and root meristem initials. Development 120, 2475–2487. doi: 10.1242/dev.120.9.2475

[ref124] Si-Ammour A. Windels D. Arn-Bouldoires E. Kutter C. Ailhas J. Meins F. Jr.. (2011). miR393 and secondary siRNAs regulate expression of the TIR1/AFB2 auxin receptor clade and auxin-related development of *Arabidopsis* leaves. Plant Physiol. 157, 683–691. doi: 10.1104/pp.111.180083, PMID: 21828251 PMC3192580

[ref125] Singh A. Bhatnagar N. Pandey A. Pandey G. K. (2015). Plant phospholipase C family: regulation and functional role in lipid signaling. Cell Calcium 58, 139–146. doi: 10.1016/j.ceca.2015.04.003, PMID: 25933832

[ref126] Sozzani R. Cui H. Moreno-Risueno M. A. Busch W. Norman J. M. V. Vernoux T.. (2010). Spatiotemporal regulation of cell-cycle genes by SHORTROOT links patterning and growth. Nature 466:128. doi: 10.1038/nature09143, PMID: 20596025 PMC2967763

[ref127] Spartz A. K. Ren H. Park M. Y. Grandt K. N. Lee S. H. Murphy A. S.. (2014). SAUR inhibition of PP2C-D phosphatases activates plasma membrane H+-ATPases to promote cell expansion in *Arabidopsis*. Plant Cell 26, 2129–2142. doi: 10.1105/tpc.114.126037, PMID: 24858935 PMC4079373

[ref128] Stepanova A. N. Robertson-Hoyt J. Yun J. Benavente L. M. Xie D. Y. Dolezal K.. (2008). TAA1-mediated auxin biosynthesis is essential for hormone crosstalk and plant development. Cell 133, 177–191. doi: 10.1016/j.cell.2008.01.047, PMID: 18394997

[ref129] Stevenson-Paulik J. Odom A. R. York J. D. (2002). Molecular and biochemical characterization of two plant inositol polyphosphate 6−/3−/5-kinases. J. Biol. Chem. 277, 42711–42718. doi: 10.1074/jbc.M209112200, PMID: 12226109

[ref130] Sun J. Bankston J. R. Payandeh J. Hinds T. R. Zagotta W. N. Zheng N. (2014). Crystal structure of a plant dual-affinity nitrate transporter. Nature 507, 73–77. doi: 10.1038/nature13074, PMID: 24572362 PMC3968801

[ref131] Sun C. H. Yu J. Q. Wen L. Z. Guo Y. H. Sun X. Hao Y. J.. (2018). Chrysanthemum MADS-box transcription factor CmANR1 modulates lateral root development via homo−/heterodimerization to influence auxin accumulation in *Arabidopsis*. Plant Sci. 266, 27–36. doi: 10.1016/j.plantsci.2017.09.017, PMID: 29241564

[ref132] Swarup K. Benkova E. Swarup R. Casimiro I. Peret B. Yang Y.. (2008). The auxin influx carrier LAX3 promotes lateral root emergence. Nat. Cell Biol. 10, 946–954. doi: 10.1038/ncb1754, PMID: 18622388

[ref133] Swarup R. Friml J. Marchant A. Ljung K. Sandberg G. Palme K.. (2001). Localization of the auxin permease AUX1 suggests two functionally distinct hormone transport pathways operate in the *Arabidopsis* root apex. Genes Dev. 15, 2648–2653. doi: 10.1101/gad.210501, PMID: 11641271 PMC312818

[ref134] Swarup R. Kramer E. M. Perry P. Knox K. Leyser H. M. Haseloff J.. (2005). Root gravitropism requires lateral root cap and epidermal cells for transport and response to a mobile auxin signal. Nat. Cell Biol. 7, 1057–1065. doi: 10.1038/ncb1316, PMID: 16244669

[ref135] Tabata R. Sumida K. Yoshii T. Ohyama K. Shinohara H. Matsubayashi Y. (2014). Perception of root-derived peptides by shoot LRR-RKs mediates systemic N-demand signaling. Science 346, 343–346. doi: 10.1126/science.1257800, PMID: 25324386

[ref136] Takahashi K. Hayashi K. Kinoshita T. (2012). Auxin activates the plasma membrane H+-ATPase by phosphorylation during hypocotyl elongation in *Arabidopsis*. Plant Physiol. 159, 632–641. doi: 10.1104/pp.112.196428, PMID: 22492846 PMC3375930

[ref137] Takatsuka H. Umeda M. (2014). Hormonal control of cell division and elongation along differentiation trajectories in roots. J. Exp. Bot. 65, 2633–2643. doi: 10.1093/jxb/ert485, PMID: 24474807

[ref138] Tan X. Calderon-Villalobos L. I. Sharon M. Zheng C. Robinson C. V. Estelle M.. (2007). Mechanism of auxin perception by the TIR1 ubiquitin ligase. Nature 446, 640–645. doi: 10.1038/nature05731, PMID: 17410169

[ref139] Tao Y. Ferrer J. L. Ljung K. Pojer F. Hong F. Long J. A.. (2008). Rapid synthesis of auxin via a new tryptophan-dependent pathway is required for shade avoidance in plants. Cell 133, 164–176. doi: 10.1016/j.cell.2008.01.049, PMID: 18394996 PMC2442466

[ref140] Trevisan S. Nonis A. Begheldo M. Manoli A. Palme K. Caporale G.. (2012). Expression and tissue-specific localization of nitrate-responsive miRNAs in roots of maize seedlings. Plant Cell Environ. 35, 1137–1155. doi: 10.1111/j.1365-3040.2011.02478.x, PMID: 22211437

[ref141] Tromas A. Braun N. Muller P. Khodus T. Paponov I. A. Palme K.. (2009). The AUXIN BINDING PROTEIN 1 is required for differential auxin responses mediating root growth. PLoS One 4:e6648. doi: 10.1371/journal.pone.0006648, PMID: 19777056 PMC2744284

[ref142] Tsukaya H. (2003). Organ shape and size: a lesson from studies of leaf morphogenesis. Curr. Opin. Plant Biol. 6, 57–62. doi: 10.1016/S1369526602000055, PMID: 12495752

[ref143] Uchida N. Takahashi K. Iwasaki R. Yamada R. Yoshimura M. Endo T. A.. (2018). Chemical hijacking of auxin signaling with an engineered auxin-TIR1 pair. Nat. Chem. Biol. 14, 299–305. doi: 10.1038/nchembio.2555, PMID: 29355850 PMC5812785

[ref144] Ulmasov T. Murfett J. Hagen G. Guilfoyle T. J. (1997). Aux/IAA proteins repress expression of reporter genes containing natural and highly active synthetic auxin response elements. Plant Cell 9, 1963–1971. doi: 10.1105/tpc.9.11.1963, PMID: 9401121 PMC157050

[ref145] Van Den Berg C. Willemsen V. Hendriks G. Weisbeek P. Scheres B. (1997). Short-range control of cell differentiation in the *Arabidopsis* root meristem. Nature 390, 287–289. doi: 10.1038/36856, PMID: 9384380

[ref146] Van Leeuwen W. Vermeer J. E. Gadella T. W. Jr. Munnik T. (2007). Visualization of phosphatidylinositol 4,5-bisphosphate in the plasma membrane of suspension-cultured tobacco BY-2 cells and whole *Arabidopsis* seedlings. Plant J. 52, 1014–1026. doi: 10.1111/j.1365-313X.2007.03292.x, PMID: 17908156

[ref147] Vidal E. A. Alvarez J. M. Gutierrez R. A. (2014). Nitrate regulation of AFB3 and NAC4 gene expression in *Arabidopsis* roots depends on NRT1.1 nitrate transport function. Plant Signal. Behav. 9:e28501. doi: 10.4161/psb.28501, PMID: 24642706 PMC4091544

[ref148] Vidal E. A. Araus V. Lu C. Parry G. Green P. J. Coruzzi G. M.. (2010). Nitrate-responsive miR393/AFB3 regulatory module controls root system architecture in *Arabidopsis thaliana*. Proc. Natl. Acad. Sci. U. S. A. 107, 4477–4482. doi: 10.1073/pnas.0909571107, PMID: 20142497 PMC2840086

[ref149] Vidal E. A. Moyano T. C. Riveras E. Contreras-Lopez O. Gutierrez R. A. (2013). Systems approaches map regulatory networks downstream of the auxin receptor AFB3 in the nitrate response of *Arabidopsis thaliana* roots. Proc. Natl. Acad. Sci. U. S. A. 110, 12840–12845. doi: 10.1073/pnas.1310937110, PMID: 23847199 PMC3732920

[ref150] Wang Y. Y. Cheng Y. H. Chen K. E. Tsay Y. F. (2018). Nitrate transport, signaling, and use efficiency. Annu. Rev. Plant Biol. 69, 85–122. doi: 10.1146/annurev-arplant-042817-040056, PMID: 29570365

[ref151] Wang J. W. Wang L. J. Mao Y. B. Cai W. J. Xue H. W. Chen X. Y. (2005). Control of root cap formation by MicroRNA-targeted auxin response factors in *Arabidopsis*. Plant Cell 17, 2204–2216. doi: 10.1105/tpc.105.033076, PMID: 16006581 PMC1182483

[ref152] Weijers D. Benkova E. Jager K. E. Schlereth A. Hamann T. Kientz M.. (2005). Developmental specificity of auxin response by pairs of ARF and aux/IAA transcriptional regulators. EMBO J. 24, 1874–1885. doi: 10.1038/sj.emboj.7600659, PMID: 15889151 PMC1142592

[ref153] Widiez T. El Kafafi El S. Girin T. Berr A. Ruffel S. Krouk G.. (2011). High nitrogen insensitive 9 (HNI9)-mediated systemic repression of root NO3- uptake is associated with changes in histone methylation. Proc. Natl. Acad. Sci. U. S. A. 108, 13329–13334. doi: 10.1073/pnas.1017863108, PMID: 21788519 PMC3156160

[ref154] Williams L. Miller A. (2001). Transporters responsible for the uptake and partitioning of nitrogenous solutes. Annu. Rev. Plant Physiol. Plant Mol. Biol. 52, 659–688. doi: 10.1146/annurev.arplant.52.1.659, PMID: 11337412

[ref155] Windels D. Vazquez F. (2011). miR393: integrator of environmental cues in auxin signaling? Plant Signal. Behav. 6, 1672–1675. doi: 10.4161/psb.6.11.17900, PMID: 22067993 PMC3329333

[ref156] Xie Q. Frugis G. Colgan D. Chua N. H. (2000). *Arabidopsis* NAC1 transduces auxin signal downstream of TIR1 to promote lateral root development. Genes Dev. 14, 3024–3036. doi: 10.1101/gad.852200, PMID: 11114891 PMC317103

[ref157] Xu P. Cai W. (2019). Nitrate-responsive OBP4-XTH9 regulatory module controls lateral root development in *Arabidopsis thaliana*. PLoS Genet. 15:e1008465. doi: 10.1371/journal.pgen.1008465, PMID: 31626627 PMC6821136

[ref158] Xu P. Chen H. Ying L. Cai W. (2016). AtDOF5.4/OBP4, a DOF transcription factor gene that negatively regulates cell cycle progression and cell expansion in *Arabidopsis thaliana*. Sci. Rep. 6:27705. doi: 10.1038/srep27705, PMID: 27297966 PMC4906354

[ref159] Xuan W. Band L. R. Kumpf R. P. Van Damme D. Parizot B. De Rop G.. (2016). Cyclic programmed cell death stimulates hormone signaling and root development in *Arabidopsis*. Science 351, 384–387. doi: 10.1126/science.aad2776, PMID: 26798015

[ref160] Xuan W. De Gernier H. Beeckman T. (2020). The dynamic nature and regulation of the root clock. Development 147:dev181446. doi: 10.1242/dev.181446, PMID: 32014866

[ref161] Yamada M. Greenham K. Prigge M. J. Jensen P. J. Estelle M. (2009). The TRANSPORT INHIBITOR RESPONSE2 gene is required for auxin synthesis and diverse aspects of plant development. Plant Physiol. 151, 168–179. doi: 10.1104/pp.109.138859, PMID: 19625638 PMC2735986

[ref162] Yin H. Li M. Lv M. Hepworth S. R. Li D. Ma C.. (2020). SAUR15 promotes lateral and adventitious root development via activating H(+)-ATPases and auxin biosynthesis. Plant Physiol. 184, 837–851. doi: 10.1104/pp.19.01250, PMID: 32651188 PMC7536663

[ref163] Yoon E. K. Yang J. H. Lim J. Kim S. H. Kim S. K. Lee W. S. (2010). Auxin regulation of the microRNA390-dependent transacting small interfering RNA pathway in *Arabidopsis* lateral root development. Nucleic Acids Res. 38, 1382–1391. doi: 10.1093/nar/gkp1128, PMID: 19969544 PMC2831332

[ref164] Yu L. H. Miao Z. Q. Qi G. F. Wu J. Cai X. T. Mao J. L.. (2014). MADS-box transcription factor AGL21 regulates lateral root development and responds to multiple external and physiological signals. Mol. Plant 7, 1653–1669. doi: 10.1093/mp/ssu088, PMID: 25122697 PMC4228986

[ref165] Yu L. H. Wu J. Tang H. Yuan Y. Wang S. M. Wang Y. P.. (2016). Overexpression of *Arabidopsis* NLP7 improves plant growth under both nitrogen-limiting and -sufficient conditions by enhancing nitrogen and carbon assimilation. Sci. Rep. 6:27795. doi: 10.1038/srep27795, PMID: 27293103 PMC4904239

[ref166] Zhang H. Forde B. G. (1998). An *Arabidopsis* MADS box gene that controls nutrient-induced changes in root architecture. Science 279, 407–409. doi: 10.1126/science.279.5349.407, PMID: 9430595

[ref167] Zhang H. Jennings A. Barlow P. W. Forde B. G. (1999). Dual pathways for regulation of root branching by nitrate. Proc. Natl. Acad. Sci. 96, 6529–6534. doi: 10.1073/pnas.96.11.6529, PMID: 10339622 PMC26916

[ref168] Zhao Y. (2012). Auxin biosynthesis: a simple two-step pathway converts tryptophan to indole-3-acetic acid in plants. Mol. Plant 5, 334–338. doi: 10.1093/mp/ssr104, PMID: 22155950 PMC3309920

[ref169] Zhao Y. Christensen S. K. Fankhauser C. Cashman J. R. Cohen J. D. Weigel D.. (2001). A role for flavin monooxygenase-like enzymes in auxin biosynthesis. Science 291, 306–309. doi: 10.1126/science.291.5502.306, PMID: 11209081

[ref170] Zheng D. C. Xia X. L. Yin W. L. (2013). Auxin promotes nitrate uptake by up-regulating AtNRT1.1gene transcript level in *Arobidopsis thaliana*. J. Beijing For. Univ. 35, 80–85. doi: 10.1099/mic.0.049619-0

[ref171] Zhuo D. Okamoto M. Vidmar J. J. Glass A. D. (1999). Regulation of a putative high-affinity nitrate transporter (Nrt2;1At) in roots of *Arabidopsis thaliana*. Plant J. 17, 563–568. doi: 10.1046/j.1365-313x.1999.00396.x, PMID: 10205909

